# Weak Noncovalent Interactions in Three Closely Related Adamantane-Linked 1,2,4-Triazole *N*-Mannich Bases: Insights from Energy Frameworks, Hirshfeld Surface Analysis, *In Silico* 11β-HSD1 Molecular Docking and ADMET Prediction

**DOI:** 10.3390/molecules27217403

**Published:** 2022-10-31

**Authors:** Lamya H. Al-Wahaibi, Mario A. Macías, Olivier Blacque, Luke S. Zondagh, Jacques Joubert, Subbiah Thamotharan, María Judith Percino, Ahmed A. B. Mohamed, Ali A. El-Emam

**Affiliations:** 1 Department of Chemistry, College of Sciences, Princess Nourah bint Abdulrahman University, Riyadh 11671, Saudi Arabia; 2 Crystallography and Chemistry of Materials, CrisQuimMat, Department of Chemistry, Universidad de Los Andes, Carrera 1 No. 18A-10, Bogotá 111711, Colombia; 3 Department of Chemistry, University of Zurich, Winterthurerstrasse 190, 8057 Zurich, Switzerland; 4 Pharmaceutical Chemistry, School of Pharmacy, University of the Western Cape, Private Bag X17, Bellville 7535, South Africa; 5 Biomolecular Crystallography Laboratory, Department of Bioinformatics, School of Chemical and Biotechnology, SASTRA Deemed University, Thanjavur 613401, India; 6 Unidad de Polímeros Y Electrónica Orgánica, Instituto de Ciencias, Benemérita Universidad Autónoma de Puebla, Val3-Ecocampus Valsequillo, Independencia O2 Sur 50, San Pedro Zacachimalpa, Puebla 72960, CP, Mexico; 7 Department of Medicinal Chemistry, Faculty of Pharmacy, Mansoura University, Mansoura 35516, Egypt

**Keywords:** adamantane, 1,2,4-triazole, single crystal X-ray, Hirshfeld surface analysis, molecular docking, ADMET prediction, 11β-HSD1 inhibitors

## Abstract

Structural analysis and docking studies of three adamantane-linked 1,2,4-triazole *N*-Mannich bases (**1**–**3**) are presented. Compounds **1**, **2** and **3** crystallized in the monoclinic *P*2_1_/*c*, *P*2_1_ and *P*2_1_/*n* space groups, respectively. Crystal packing of **1** was stabilized by intermolecular C-H⋯O interactions, whereas compounds **2** and **3** were stabilized through intermolecular C-H⋯N, C-H⋯S and C-H⋯π interactions. The energy frameworks for crystal structures of **1**–**3** were described. The substituent effect on the intermolecular interactions and their contributions were described on the basis of Hirshfeld surface analyses. The 11β-hydroxysteroid dehydrogenase type 1 (11β-HSD1) inhibition potential, pharmacokinetic and toxicity profiles of compounds **1**–**3** were determined using *in silico* techniques. Molecular docking of the compounds into the 11β-HSD1 active site showed comparable binding affinity scores (−7.50 to −8.92 kcal/mol) to the 11β-HSD1 co-crystallized ligand **4YQ** (−8.48 kcal/mol, 11β-HSD1 IC_50_ = 9.9 nM). The compounds interacted with key active site residues, namely Ser170 and Tyr183, via strong hydrogen bond interactions. The predicted pharmacokinetic and toxicity profiles of the compounds were assessed, and were found to exhibit excellent ADMET potential.

## 1. Introduction

**Figure 1:**
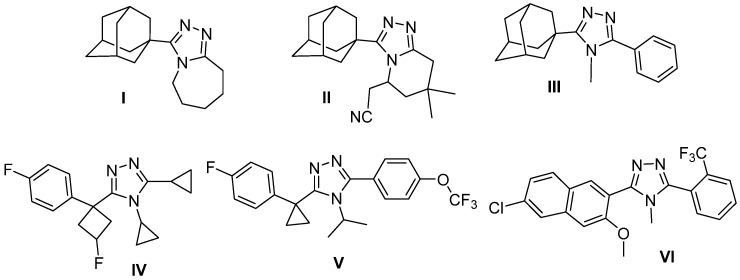
The structures of the adamantyl-1,2,4-triazole (I–III) and des-adamantyl-1,2,4-triazole (IV–VI) 11β-HSD1 inhibitors.

Adamantane-based derivatives, which have long been identified for their diverse pharmacological activities [1,2,3,4], and several adamantane-based drugs, are currently used as efficient chemotherapies as antiviral [5,6,7], anti-TB [8,9] and anticancer agents [10,11,12]. The dipeptidyl peptidase IV (DPP-IV) adamantane-based drugs, saxagliptin [13] and vildagliptin [14], are currently used as oral hypoglycemic agents for the treatment of type 2 diabetes. The adamantane-linked 1,2,4-triazole derivatives I, II and III (Figure 1) were further discovered to be potent inhibitors of 11β-hydroxysteroid dehydrogenase type 1 (11β-HSD1) [15,16]. 11β-HSD1 is an NADPH-dependent reductase that converts inactive cortisone into the active glucocorticoid cortisol. Cortisol stimulates gluconeogenesis through upregulation of enzymes, such as phosphoenolpyruvate carboxykinase and glucose-6-phosphatase; in adipose tissues, cortisol promotes adipogenesis and lipolysis. Thus, 11β-HSD1 regulates intracellular cortisol levels, and has been implicated in a number of metabolic sequela of increased glucocorticoid tone, such as visceral adiposity, elevated blood pressure, elevated fasting glucose and dyslipidemia [17]. In contrast, 11β-hydroxysteroid dehydrogenase type 2 (11β-HSD2) is an NAD-dependent dehydrogenase that catalyzes the conversion of cortisol to cortisone. 11β-HSD2 is expressed in cells that contain the mineralocorticoid receptors, and protects mineralocorticoids from illicit occupation by cortisol. Inhibition of 11β-HSD2 is known to result in hypokalemia, sodium retention, and hypertension. Consequently, the development of selective 11β-HSD1 inhibitors could be an important therapy for non-insulin-dependent diabetes, hyperglycemia, obesity, insulin resistance, hyperlipidemia, hypertension and other symptoms that are associated with excessive body cortisol [18,19,20]. In addition, the des-adamantyl 1,2,4-triazole derivatives IV [21], V [22] and VI [23], are currently under clinical investigations as 11β-HSD1 inhibitors for the treatment of type 2 diabetes and obesity (Figure 1). According to experimental and molecular docking studies for the identification of chemical features of 11β-HSD1 inhibitors, a combination of an adamantane cage- and 1,2,4-triazole or other azole moieties could result in potent 11β-HSD1 inhibitors [15,16,24,25,26,27,28,29].

In continuation with ongoing interest in the structural properties [30,31,32,33,34,35] and biological applications of adamantane-based derivatives [35,36,37,38,39], we report herein on the molecular structure insights, Hirshfeld surface analysis and pairwise interaction energies of three adamantane-linked 1,2,4-triazole *N*-Mannich bases, namely ethyl 4-[(3-adamantan-1-yl)-4-ethyl-5-thioxo-4,5-dihydro-1*H*-1,2,4-triazol-1-yl)methyl]piperazine-1-carboxylate (**1**), 5-(adamantan-1-yl)-4-ethyl-2-[(4-(pyridin-2-yl)piperazin-1-yl)methyl]-2,4-dihydro-3*H*-1,2,4-triazole-3-thione (**2**) and 5-(adamantan-1-yl)-4-allyl-2-[(4-(pyridin-2-yl)piperazin-1-yl)methyl]-2,4-dihydro-3*H*-1,2,4-triazole-3-thione (**3**), which were proven to possess marked hypoglycemic activity [39]. Molecular docking analyses at the 11β-HSD1 active site were also performed, in order to predict the potential 11β-HSD1 binding affinity and binding interactions of the compounds. The key active site residues, which establish noncovalent interactions with the title compounds, were identified to understand the binding affinity and the effect of their substituents.

## 2. Results and Discussion

### 2.1. Synthesis and Crystallization

**Scheme 1:**
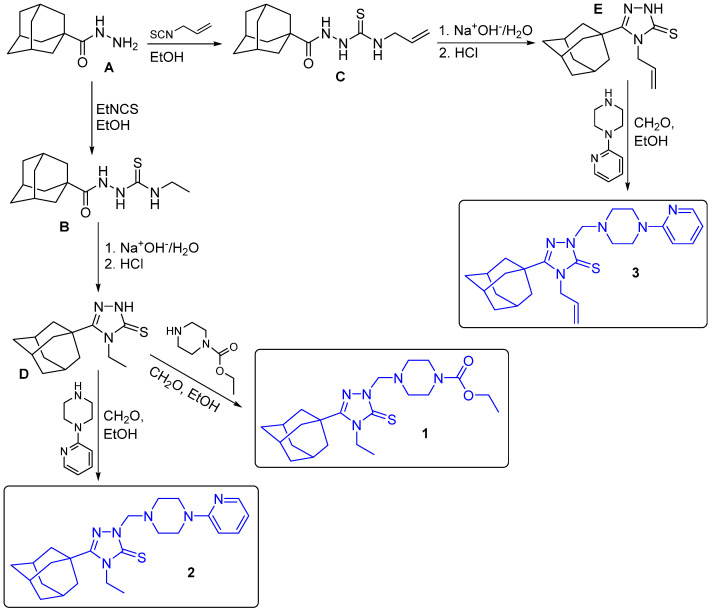
Synthesis of compounds **1**, **2** and **3**.

Compounds **1**, **2** and **3** were prepared, beginning with adamantane-1-carbohydrazide **A**, following previously reported procedures [39]. Scheme 1 summarizes the reaction sequences that lead to the target compounds and their intermediates **B**, **C**, **D [40]** and **E** [41]. Suitable single crystals of compounds **1**, **2** and **3** were obtained by slow evaporation of solutions of the compounds in EtOH/CHCl_3_ (1:2, *v*/*v*) at room temperature.

### 2.2. Description of Molecular and Crystal Structures

**Figure 2:**
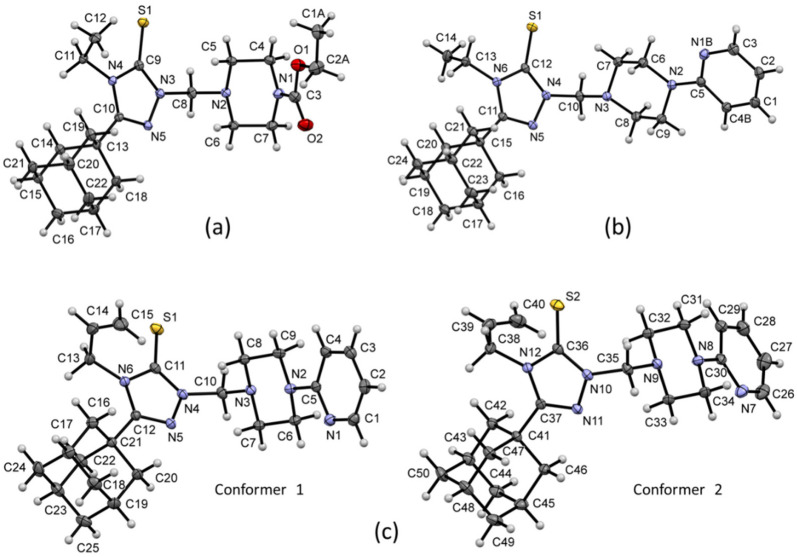
Molecular structures of (**a**) **1**, (**b**) **2** and (**c**) **3**, with anisotropic thermal vibration ellipsoids drawn at the 50% probability level. The hydrogen atoms are shown as spheres of arbitrary radius.

Crystal data, and structure refinement details of compounds **1**–**3** are presented in Table 1, and their ORTEP plots are shown in Figure 2. There was a difference between compounds **1** and **2** in the substituent that was attached to one of the N atoms of the piperazine moiety (pyridine or ethoxycarbonyl). An allyl group was attached to the triazole ring in compound **3**, while the corresponding position was occupied by an ethyl group in compounds **1** and **2**. Single-crystal X-ray diffraction analyses revealed that all three compounds crystallized in the monoclinic system. Compound **2** crystallized with the non-centrosymmetric space group *P*2_1_, while compounds **1** and **3** crystallized with the centrosymmetric space groups *P*2_1_/*c* and *P*2_1_/*n*, respectively. Moreover, the asymmetric unit of compound **3** consisted of two crystallographically independent molecules, and the corresponding unit only had a single molecule for the other two compounds.

**Figure 3:**
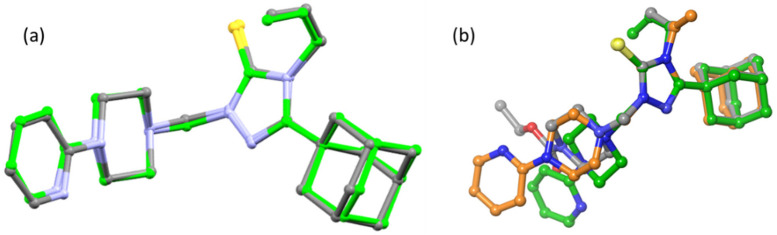
(**a**) Structural superimposition of molecules A (grey) and B (green) of compound **3**; (**b**) structures of **1** (grey), **2** (orange) and **3** (green) superimposed with respect to the central triazole ring atoms.

As shown in Figure 3a, the conformations of molecules A and B superimposed very well. Additionally, no apparent higher crystallographic symmetry was detected from the PLATON program [42], which confirmed the presence of two molecules in the asymmetric unit. Furthermore, we superimposed structures of compounds **1**–**3** (considering only major disordered components and molecule A of **3**), with respect to the central triazole core, revealing structural deviations around piperazine-containing fragments. The adamantane core and ethyl groups superimposed very well (Figure 3b). The dihedral angle that formed between the mean planes of the adamantane moiety and central triazole ring was 42–48° in these structures. However, the relative orientation between mean planes of piperazine and triazole rings was wider in structure **3** (82.82° in mol A and 78.17° in mol B) than in structures **1** (71.01°) and **2** 65.22°). Furthermore, Cremer and Pople puckering parameters suggested that six-membered rings of the adamantane cage and piperazine ring exhibited a typical chair conformation in all three structures [43].

#### 2.2.1. Crystal Packing of Compound **1** and Interactions

**Figure 4:**
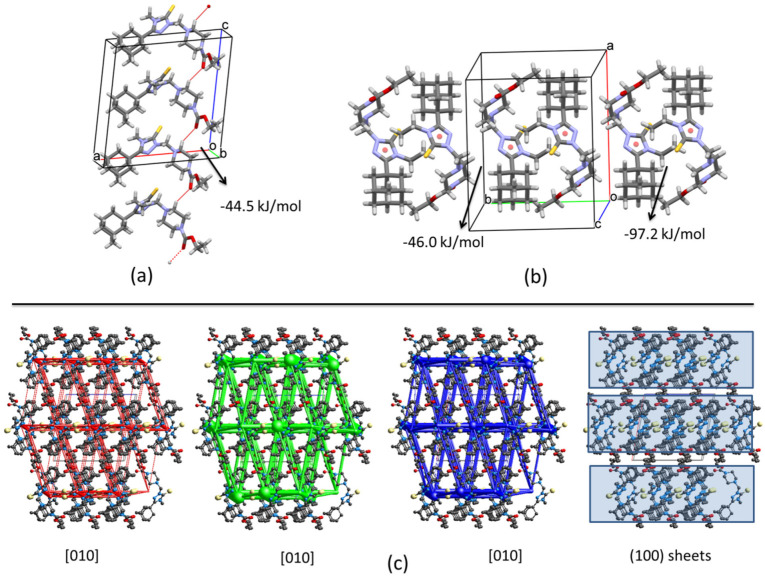
Crystal structure of compound **1** showing the (**a**) C-H⋯O hydrogen bonds, and (**b**) pairs of inversion-related molecules along the [010] direction. (**c**) Energy framework diagrams for electrostatic (red) and dispersion (green) contributions to the total interaction energies (blue). Moreover, the crystal packing showing the (100) sheets is depicted.

The selected intra- and intermolecular geometries for structures **1**–**3** are listed in Table 2. In **1**, two weak intramolecular C–H⋯O interactions formed, and both oxygens were involved as acceptors. Apparently, the most important intermolecular interaction corresponded to the C5-H5B⋯O2^i^ (symmetry code: (i) x,1/2 − y,1/2 + z) hydrogen bonds, which connected molecules along the [001] direction (Figure 4a and Table 2). This interaction was possible, due to the presence of the unshared pair of electrons over the oxygen atom in the ethoxycarbonyl group, and the slightly acidic tendency of H5B. In fact, this hydrogen bond was very short (2.40 Å), and the molecular centroids (mean atomic positions) between molecules along this direction had a distance of 7.25 Å. The pairwise interaction energy between these molecules was calculated to be −44.5 kJ/mol. As observed in Table 3, dispersion forces were the principal contributors to the total energy, which allowed for the imagining that the C5-H5B⋯O2^i^ hydrogen bond was not the only one responsible for the attraction. Between molecules along the [001] direction, sulfur atoms were in the middle; thus, even the adamantane rings could have contributed to the interaction. The analysis of the supramolecular interactions from the hydrogen bonds suggests that C5-H5B⋯O2^i^ are the most important in the formation of the solid.

However, from the calculated pairwise interactions energies, there was another interaction with a total energy of −97.2 kJ/mol which corresponded to the interaction between a pair of inversion-related molecules (Figure 4b). Apart from the interaction between the terminal methyl group and the triazole-sulfanyl ring (~3.14 Å), the interaction had a dispersion value of −151.9 kJ/mol, which was the highest contribution from dispersion energies for this compound (Table 3). From an energetic perspective, it could be possible to make reference to this pair as “dimers”, which had a distance of 4.72 Å between their molecular centroids. Considering that, two neighboring “dimers” had a pairwise interaction energy of −46.0 kJ/mol along the [010] direction. Combining this perception with the C5-H5B⋯O2^i^ hydrogen bonds, it is possible to conclude that the crystal structure of 1 formed molecular sheets that stacked along the [100] direction, leaving only weak interactions between sheets (Table 3).

Computed energies between molecular pairs are represented using cylinders joining the centroids (molecular center of mass) of the molecules, with a radius proportional to the magnitude of the interaction, managing a minimal cut-off of 5 kJ/mol. Figure 4c shows the energy framework diagrams for pairs of molecules for separate, electrostatic (red) and dispersion (green) contributions to the total nearest-neighbor pairwise interaction energies (blue) for compound **1**. The energy frameworks showed strong intermolecular interactions that formed the sheets, and also the high importance of dispersion forces in the formation of the crystal.

**Figure 5:**
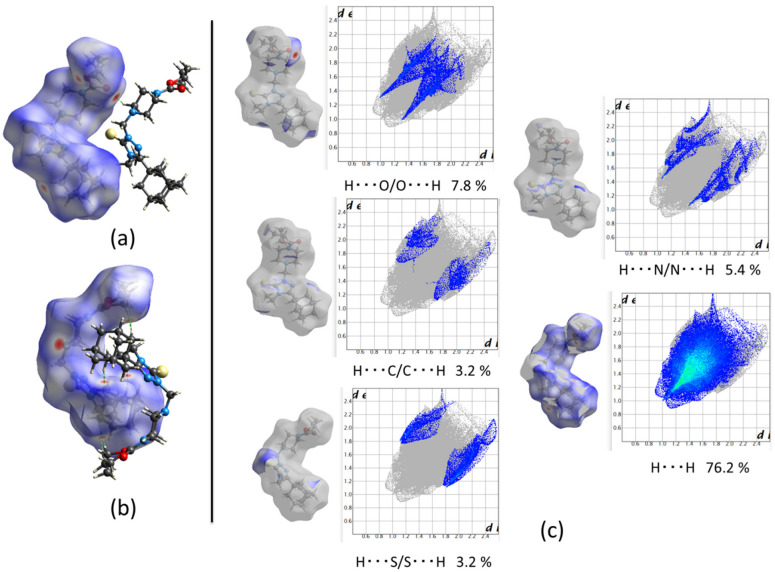
Hirshfeld surface mapped over *d*_norm_ showing the (**a**) C-H⋯O hydrogen bonds, and (**b**) the high packing between inversion-related molecules and their H⋯H interactions. (**c**) 2D fingerprint plots and relative contributions (%) to the Hirshfeld surface area for the various close intermolecular contacts in compound **1**.

The short C5-H5B⋯O2^i^ hydrogen bonds are visible on the Hirshfeld surface (HS) map (Figure 5a). As previously discussed, pairs of inversion-related molecules represented the strongest interactions in the crystal. Figure 5b shows that the closeness of these molecules induced non-covalent H⋯H interactions, which contributed to the high dispersion energy value (Table 3). The strong attraction between these molecules was observed in the form of the HS, which showed a high degree of packing. Figure 5c shows the 2D (dimensional) fingerprints plots. Despite their low contribution to the HS (7.8%), C5-H5B⋯O2^i^ hydrogen bonds played an important role in the formation of the (100) sheets. Other interactions, such as H⋯S/S⋯H (3.2%), H⋯C/C⋯H (3.2%) and H⋯N/N⋯H (5.4%), contributed poorly. However, interesting results showed the high contribution to the HS from non-covalent H⋯H interactions (76.2%), which is perfectly consistent with the fact that dispersion forces were the highest contributors to the formation of the crystal, according to the energy frameworks.

#### 2.2.2. Crystal Packing of Compound **2** and Interactions

**Figure 6:**
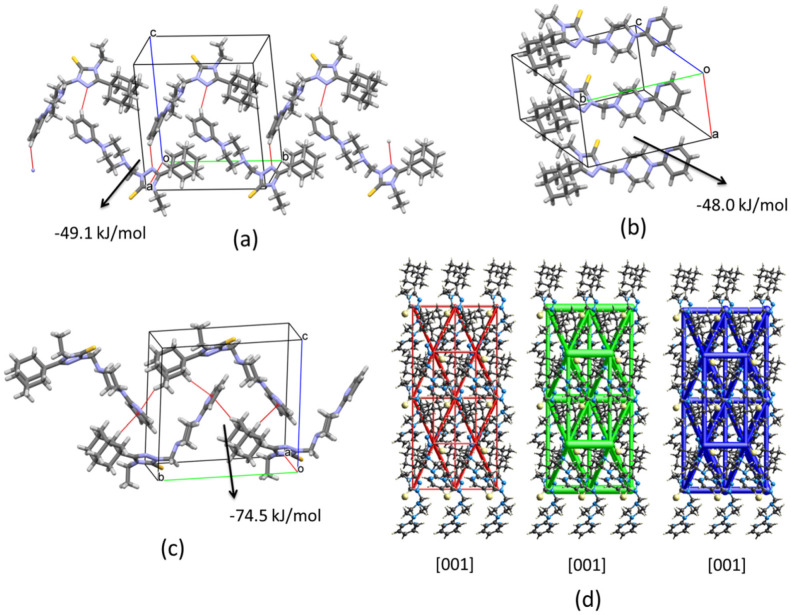
Crystal structure of compound **2** showing the (**a**) C-H⋯N hydrogen interactions, (**b**) molecules oriented along [100] interacting via dispersion forces, (**c**) C-H⋯π interactions, and (**d**) energy framework diagrams for electrostatic (red) and dispersion (green) contributions to the total interaction energies (blue).

The crystal structure of compound **2** had notable differences compared with **1**. These differences were the consequence of the presence of one pyridine group instead of the ethoxycarbonyl group. Clearly, the observed C-H⋯O hydrogen bonds in **1** were not possible in the crystal growth of **2**. In fact, the crystal structure lacked short hydrogen interactions, and the only possibility for such contacts was due to the triazole ring. Long (weak) C1-H1A⋯N5^ii^ (symmetry code: (ii) 2 − x,−1/2 + y,1 − z) hydrogen interactions were detected that connected molecules in chains that ran along the [010] direction (Figure 6a). This interaction had pairwise interaction energy with a value of −49.1 kJ/mol, and higher contribution from the dispersion term (Table 3). Another interaction with similar energy was observed along the [100] direction (−48.0 kJ/mol), without evidence of classic hydrogen bonds and higher contributions from dispersion forces (Figure 6b and Table 3). However, the C13-H13B⋯S1^iii^ (symmetry code: (iii) 1 − x,1/2 + y,2 − z) interaction was not discarded, considering previous reports [44]. Despite the molecular closeness of this last interaction, i.e., 6.34 Å between molecular centroids, this was not the strongest contact in the crystal. The orientation of the molecules due to the packing allowed for the formation of a combination of C21-H21A⋯Cg1^iv^ and C23-H23A⋯Cg1^v^ (symmetry codes: (iv) 1 − x,1/2 + y,1 − z; (v) x,1 + y,z; Cg1 was the centroid of the C1/N1B ring, that is, the pyridine group) interactions along the [010] direction, and a pairwise interaction energy of −74.5 kJ/mol. Obviously, this interaction also had a higher contribution from dispersion forces (Figure 6c and Table 3). Other interactions complemented the formation of the crystal with lower energies that, altogether with the already mentioned interactions (Table 3), resulted in an energetic topology with a more 3-dimensional tendency compared with **1**. The energy frameworks observed in Figure 6d show that tendency.

**Figure 7:**
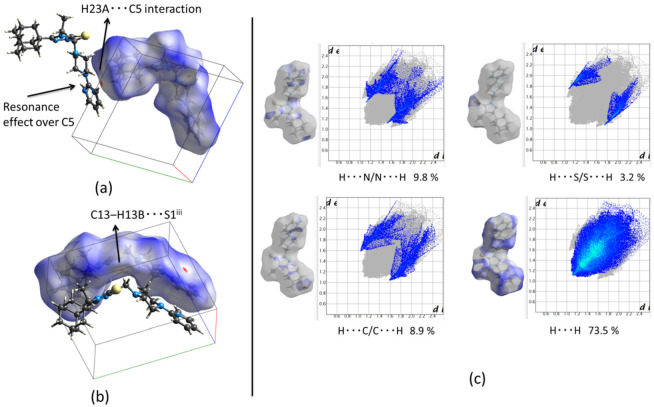
Hirshfeld surface mapped over *d*_norm_ showing the (**a**) H23A⋯C5, and (**b**) C13-H13B⋯S1 hydrogen interactions. (**c**) 2D fingerprint plots and relative contributions (%) to the Hirshfeld surface area for the various close intermolecular contacts in compound **2**.

Hirshfeld surface (HS) maps allowed us to detect the C-H⋯π interaction which corresponded to the C23-H23A⋯Cg1^iv^ (symmetry code: (iv) x, 1 + y, z) contact. However, a closer inspection shows that the red spot was oriented more towards an H23A⋯C5 interaction, which is perfectly possible due to the neighboring nitrogen atoms and the resonance effect (Figure 7a). The HS mapped over *d*_norm_ allowed corroboration of the C13-H13B⋯S1^iii^ (symmetry code: (iii) 1 − x,1/2 + y,2 − z) interaction that was previously discussed (Figure 7b). Contributions to the total HS area from interactions such as H⋯S/S⋯H (3.2%), H⋯C/C⋯H (8.9%) and H⋯N/N⋯H (9.8%) were low. Similarly, to **1**, the crystal structure was built mainly by dispersion forces, and the high contribution from non-covalent H⋯H contacts (73.5%) was consistent with this observation (Figure 7c).

#### 2.2.3. Crystal Packing of Compound **3** and Interactions

**Figure 8:**
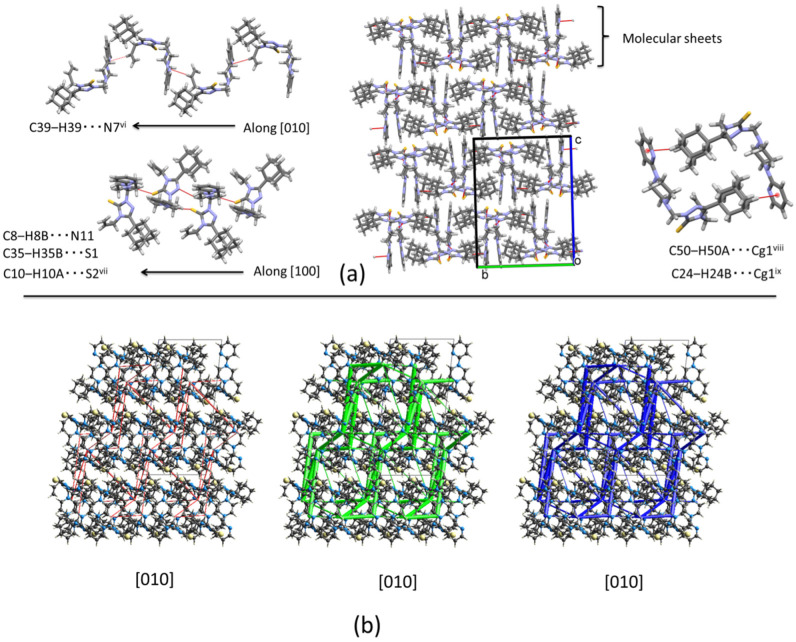
Molecular packing of compound **3** showing the (**a**) sheets stacked along the [001] direction, and (**b**) energy framework diagrams for electrostatic (red) and dispersion (green) contributions to the total interaction energies (blue).

Figure 2 shows that the molecular structure of compound **3** was very similar to the structure of **2**. The only difference was observed in the length of the substituent fragment (allyl) in the triazole ring, which changed from an ethyl to a propylene group. As expected, the supramolecular structure was characterized by the absence of short hydrogen interactions. However, the presence of the propylene fragment completely altered the molecular assembly compared with **2**. In this case, a combination of weak interactions, C39-H39⋯N7^vi^ (involving the triazole ring and the propylene fragment)/ C8-H8B⋯N11 (involving the triazole and piperazine rings) (symmetry code: (vi) 3/2 − x, 1/2 + y, 3/2 − z), and C35-H35B⋯S1/C10-H10A⋯S2^vii^ (symmetry code: (vii) 1 + x, y, z), involving the sulfur atoms and the methylene bridge built molecular sheets that were stacked along the [001] direction (Figure 8a). Additionally, a combination of C50-H50A⋯Cg1^viii^ and C24-H24B⋯Cg1^ix^ (symmetry codes (viii) 1/2 − x, 1/2 + y, 3/2 − z; (ix) 1/2 − x, −1/2 + y, 3/2 − z) interactions helped in the formation of the sheets (Table 2 and Figure 8a). The energies involved in the formation of the sheets, and correlated to these interactions, were −25.2 kJ/mol, −12.3 kJ/mol, and −12.3 kJ/mol. However, between sheets, pairwise interaction energies on the order of −38.1 kJ/mol and −20.6 kJ/mol were acting to keep the molecules assembled along the [001] direction. Dispersion forces were predominant in the three dimensions which were observed in the energy frameworks (Figure 8b).

**Figure 9:**
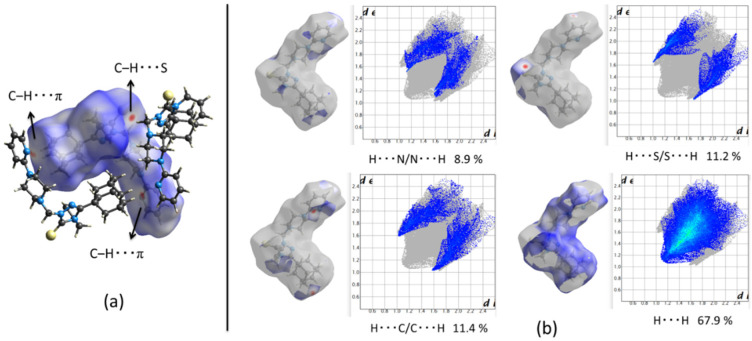
Hirshfeld surface mapped over d_norm_ showing the (**a**) C-H⋯π, and C-H⋯S hydrogen interactions. (**b**) 2D fingerprint plots and relative contributions (%) to the Hirshfeld surface area for the various close intermolecular contacts in compound **3**.

Hirshfeld surface maps allowed for corroboration of the observed interactions. Figure 9 shows the HS mapped over *d*_norm_, and the corresponding 2D fingerprint plots for compound 3. Interestingly, the H⋯S/S⋯H contributions to the total HS area were higher (11.2%) compared with **1** and **2**, showing that sulfur atoms play an important role in the formation of the crystal. This behavior could be correlated with a more nucleophilic tendency over the S atom in **3**. Other contributions, such as H⋯C/C⋯H (11.4%), H⋯N/N⋯H (8.9%), and the non-covalent H⋯H contacts (67.9%), were similar to those observed for **1** and **2**. In the last case, the dominance of the dispersion forces in the solid were corroborated.

### 2.3. Molecular Docking Analysis

**Figure 10:**
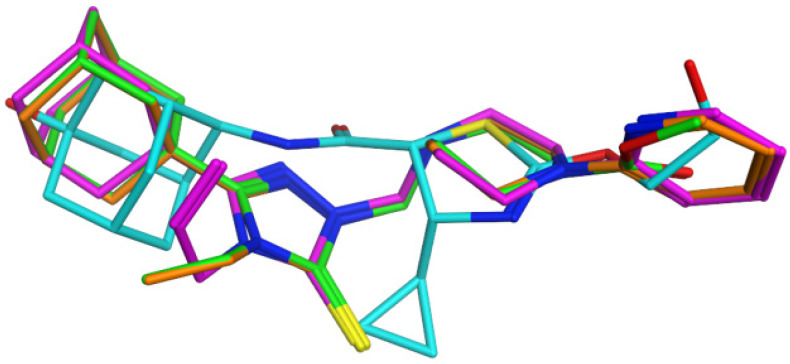
The overlaid binding conformations of **4YQ** (cyan), **1** (green), **2** (orange) and **3** (pink) are shown.

*In silico* drug design methods, such as molecular docking, are recognized to play an essential role in the development of novel therapeutics. In previous studies [24,25,28], compounds containing structural motifs, such as adamantane and triazole moieties, exhibited potent 11β-HSD1 inhibitory activities. Compounds **2** and **3** have previously been evaluated for their in vivo hypoglycemic activity. The compounds exhibited similar hypoglycemic activity, at 10 mg/kg, in streptozotocin-induced diabetic rats, compared to the approved type 2 diabetes therapeutic agent gliclazide [39]. Therefore, to explore the potential hypoglycemic mechanism of action of the compounds, we conducted molecular docking of the crystallized compounds **1**, **2** and **3**, in order to determine their potential as 11β-HSD1 inhibitors. The potential 11β-HSD1 inhibitory activity of compound **1** was also investigated due to its chemical structure similarity to compounds **2** and **3**, as well as for containing the adamantane and triazole pharmacophores known for their importance in 11β-HSD1 inhibitory activity. Therefore, we conducted molecular docking of the crystallized compounds **1**, **2** and **3**, in order to determine their potential as 11β-HSD1 inhibitors. The X-ray 11β-HSD1 protein (PDB ID: *4C7J*) was utilized for its reliability to reproduce binding poses of known 11β-HSD1 inhibitors [25], as well as the co-crystallized inhibitor 4-cyclopropyl-*N*-(trans-5-hydroxy-2-adamantyl)-2-(2-hydroxyethoxy)-thiazole-5-carboxamide (**4YQ**, 11β-HSD1 IC_50_ = 9.9 nM) [28], which contains similar structural features as compounds **1**–**3**. The compounds were docked within 11β-HSD1, and exhibited promising binding affinity scores and binding interactions with important active site residues. The docked ligands exhibited similar conformations and positioning to **4YQ** within the active site (Figure 10).

**Figure 11:**
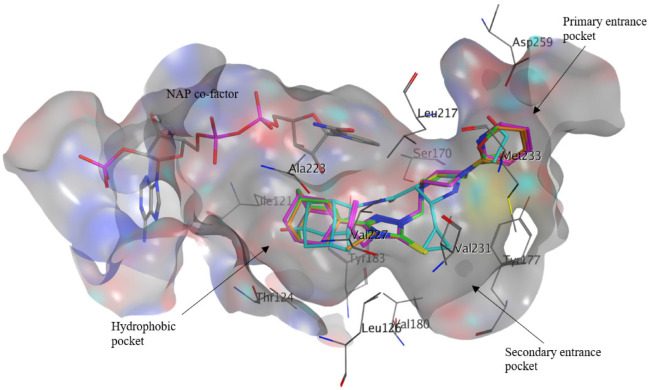
The overlaid binding orientations of **4YQ** (cyan), compound **1** (green), compound **2** (orange) and compound **3** (pink) within the 11β-HSD1 active site are shown. The NAP cofactor can be observed at the top left of the figure, forming part of the hydrophobic pocket.

**Figure 12:**
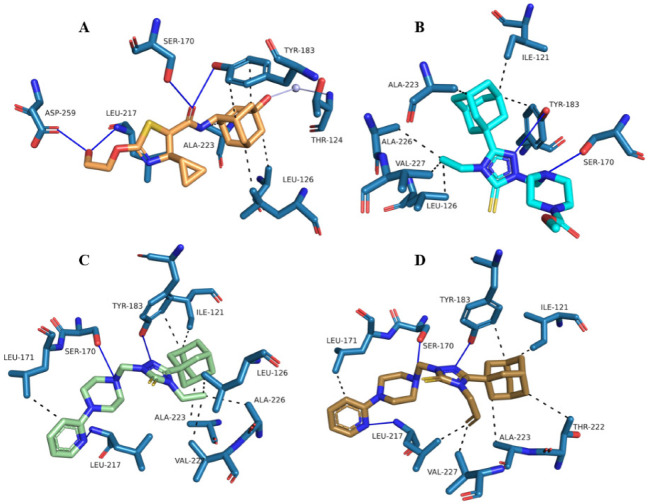
The binding orientations and interactions of **4YQ** (**A**), compound **1** (**B**), compound **2** (**C**) and compound **3** (**D**) within the 11β-HSD1 (PDB: *4C7J*) active site. White spheres represent water molecules; white solid lines represent water bridge interactions; blue solid lines represent hydrogen bonds and black dashed lines represent hydrophobic interactions.

The adamantane moiety of **4YQ** was situated deep within the hydrophobic pocket of the active site that contained residues Val180, Ile121, Thr124, Leu126, Ala223 and cofactor NAP. The substituted thiazole moiety and carboxamide linker were positioned within the midsection of the active site that contained key catalytic residues Ser170 and Tyr183. The 4-cyclopropyl substitution of **4YQ** stretched into the secondary entrance of the protein-containing residues Tyr177, Val231, Val227 and Met233; finally, the 2-hydroxyethoxy substitution stretched out towards the primary entrance of the active site that contained residues Asp259 and Leu217 (Figure 11 and Figure 12).

NAD(P)H (NAP) cofactor acts as a catalyst in the conversion of inactive cortisone into active cortisol, and studies have found that 11β-HSD1 activity is dependent on the presence of NAD(P)H [45,46]. **4YQ** obtained a binding affinity score of −8.48 kcal/mol (Table 4), and displayed multiple hydrophobic interactions within both the hydrophobic pocket and the secondary entrance. The co-crystallized ligand exhibited strong hydrogen bond interactions between the adamantyl hydroxyl group and Thr124, the 2-hydroxyethoxy substitution and residues Asp259 and Leu217, and the thiazole moiety and key catalytic residues Tyr183 and Ser170. Ser170 and Tyr183 are located within the catalytic active site of the protein. The two residues anchor the substrate of 11β-HSD1 within the catalytic active site, and in turn, play a crucial role in the proton transfer between the substrate and NAD(P)H cofactor [47,48,49]. **4YQ** displayed multiple water bridge interactions within both the hydrophobic pocket and the primary entrance of the protein, showing the importance of water molecules within active sites for improved pose accuracy of molecular docking predictions [50].

The docked ligands (compounds **1**, **2** and **3**) obtained binding affinity scores of −8.46 kcal/mol, −8.92 kcal/mol and −7.50 kcal/mol, respectively (Table 4). It can be observed in Figure 10 and Figure 11 that the docked ligands’ adamantane moieties are buried deeper within the hydrophobic pocket, resulting in stronger hydrophobic interactions when compared to **4YQ**. The substituted triazole moieties adopted a similar position and conformation to **4YQ**’s carboxamide linker, resulting in a hydrogen bond interaction with the key catalytic residue Tyr183. The piperazine rings of the docked compounds were situated in a similar position to the substituted thiazole moiety of **4YQ**, and interacted with the key catalytic residue Ser170 via a hydrogen bond interaction. The 5-thione substitution on the triazole moiety for all of the docked ligands was situated deep within the secondary entrance of the protein. However, no binding interactions were observed, as the secondary entrance pocket predominantly consisted of hydrophobic and proton-accepting residues.

The 4-ethyl substitutions of **1** and **2**, and the 4-propene of **3** conjugated to the triazole moiety, were unable to access the secondary entrance pocket of the active site; this resulted in hydrophobic interactions with the residues on the edge of the hydrophobic pocket. The replacement of the 4-ethyl substitution for a 4-propene substitution on the triazole moiety resulted in a significantly reduced binding affinity score for **3**, with no additional binding interactions when compared to **2**. The ethoxycarbonyl substitution on the piperazine moiety of **1** exhibited binding interactions between the unsaturated oxygen and Leu217. A proton-donating hydrogen bond interaction between the hydroxyl group of the hydroxyethoxy substitution of **4YQ** and Asp259 was observed (Figure 12). Interestingly, no interaction between the ethyloxycarbonyl substitution of **1** and Asp259 was observed, even though the methyl moiety was in close proximity to the residue (Figure 11). The oxygen of a hydroxyl group is more electronegative than the carbon of a methyl group; hence, less energy is required for the hydroxyl group of **4YQ** to donate its hydrogen to Asp259 and become protonated, compared to the methyl group of **1**. Consequently, future 11β-HSD1 inhibitors should contain highly electronegative proton-donating groups, in order to interact with the residue Asp259. Compounds **2** and **3** contain a pyridin-2-yl substitution on the piperazine moiety. The nitrogen of the pyridin-2-yl for both docked ligands exhibited a hydrogen bond interaction with Leu217. Aromatic stacking interactions (Tables S4, S6 and S8) were formed between the 2-N group on the triazole moiety and Tyr183, for all the compounds. Compounds **2** and **3** also formed aromatic stacking interactions between the pyridin-2-yl moiety and Tyr177.

The docked ligands exhibited similar binding interactions when compared to **4YQ**. The investigated compounds interacted with the hydrophobic pocket residues that are in close proximity with the NAP cofactor, and interacted with key catalytic residues Ser170 and Tyr183 through strong hydrogen bond interactions. Compound **1** obtained a similar binding affinity score to **4YQ**, whereas **2** exhibited a significantly improved binding affinity score when compared to the co-crystallized ligand. The addition of a 4-propene substitution (**3**) over a 4-ethyl substitution (**1** and **2**) on the triazole moiety led to no additional binding interactions, and an inferior binding affinity score. The detailed visual representations of binding interactions of compounds **4YQ**, **1**, **2** and **3** within the *4C7J* active site are shown in Figures S1–S4, respectively, and their detailed binding interactions with *4C7J* active site residues are shown in Tables S1–S8.

### 2.4. ADMET Analysis

Three web-based ADMET analysis tools, namely ProTox-II, SwissADME and admetSAR, were employed to predict the ADME and toxicity profiles of the compounds. Protox-II web-based analysis tool was employed to predict the toxicity of the compounds (Table 5 and Figures S5–S10). Compound **1** was predicted to have an oral toxic dose of 1000 mg/kg, whereas compounds **2** and **3** were predicted to have an oral toxic dose of 162 mg/kg. All of the compounds were predicted to be in oral toxicity class IV. The oral toxic dose is equivalent to the median lethal dose (LD_50_, mg/kg). Oral toxicity classes III and IV for the ProTox-II online prediction tool are equivalent to LD_50_ ranges of 50 mg/kg < LD_50_ ≤ 300 mg/kg and 300 mg/kg < LD_50_ ≤ 2000 mg/kg, respectively. The compounds were further assessed for their predicted hepatotoxicity, carcinogenicity, immunotoxicity, mutagenicity and cytotoxicity. No toxicity was predicted by the compounds against any of the target classes, other than for compounds **2** and **3**, which were predicted to be carcinogenic. The pyridin-2-yl substitution on the triazole moiety was the only difference between **1** and **2**, and the toxic dose value was constant between **2** and **3**. Consequently, it can be deduced that the pyridin-2-yl fragment plays a role in the increased predicted toxicity of compounds **2** and **3**.

The STopTox online acute toxicity prediction tool predicts the commonly known “6-pack” assays (Table 5 and Figures S11–S13) that are required by various drug regulatory agencies to evaluate multiple aspects of acute toxicities in humans, using machine learning (ML) models [51]. The “6-pack” assay for acute toxicity includes acute oral, dermal and inhalation toxicity, skin and eye irritation and corrosion as well as skin sensitization. The compounds were predicted to have no acute dermal toxicity; however, they were predicted to contain acute oral toxicity, as well as eye irritation and corrosion properties. Compounds **2** and **3** were predicted to contain acute inhalation toxicity and skin irritation properties. It can be observed in Figures S11–S13 that the replacement of the ethoxycarbonyl substitution with a pyridin-2-yl on the piperazine moiety resulted in an increase in the piperazine moiety’s predicted fragment contribution to acute inhalation toxicity. The pyridin-2-yl was also predicted to have a direct fragment contribution to skin irritation and corrosion. Compound **3** was predicted to cause skin sensitization and or allergic skin reactions. The addition of the 4-propene can be observed in Figure S12 to have a direct fragment contribution to skin sensitization. The correlation between an increase in toxicity and the addition of a pyridin-2-yl and/or propene substitution corresponds with the toxicity prediction results that were obtained with the Protox-II online toxicity prediction tool. The SwissADME web-based ADME prediction tool was employed to predict the ADME properties of the compounds (Table 6 and Figures S8–S10). The compounds were predicted to have high absorption, to not be P-glycoprotein (P-gp) substrates, to obtain a bioavailability score of 0.55, and to pass all of the Lipinski’s rule of five criteria. The Lipinski rule of five refers to molecular properties that are important for a therapeutic agent’s pharmacokinetics [52]. The bioavailability score (Abbot bioavailability score) of 0.55 means that the compounds had a predicted probability of 55% to be orally bioavailable [53]. Compounds **2** and **3** were predicted to cross the blood–brain barrier (BBB). The 5-thione substitution on the triazole moiety was shown as a Brenk [54] structural alert as a possible toxic thiocarbonyl group, and **2**′s propene substitution on the triazole moiety was also a Brenk structural alert as a possible toxic isolated alkene fragment. The toxicity prediction of the isolated alkene fragment correlated with the toxicity predictions that were obtained from both the ProTox-II and STopTox online toxicity prediction tools. The compounds were, in general, predicted to inhibit the majority of the human cytochrome P450 (CYP-450) enzymes.

The AdmetSAR online ADMET prediction tool was employed to further predict and correlate the ADMET results (Table 5 and Table 6 and Figures S5–S7) that were obtained by ProTox-II and SwissADME. The compounds were predicted to be human orally bioavailable, be absorbed across the human intestine, and to permeate across the BBB. The BBB permeability predictions correlated with the SwissADME BBB permeability predictions, except for compound **1**. Adamantane scaffolds have previously been utilized as a lipophilic carrier to transport molecules across the BBB and into the central nervous system (CNS) [55]. Therefore, admetSAR BBB permeability predictions can be considered the more accurate between the two online ADME prediction tools. None of the compounds were predicted to be human colorectal-adenocarcinoma-cell (caco-2) permeable. Compounds **2** and **3** were predicted to be a P-gp substrate and a P-gp inhibitor, respectively. The addition of the predicted toxic fragment propene substituted on the triazole moiety plays a crucial role in the predicted inhibition of P-gp. No carcinogenic, Ames mutagenesis or eye irritation and corrosion structural properties were predicted to be contained by the compounds. The Ames mutagenesis results corresponded to the mutagenicity results that were obtained by ProTox-II, whereas the carcinogenicity and eye irritation and corrosion results did not correspond to the results obtained by ProTox-II and STopTox, respectively. STopTox predicted that all of the compounds would cause eye irritation and corrosion structural properties. ProTox-II predicted **2** and **3** to be carcinogenic. AdmetSAR’s carcinogenicity model obtained a higher non-carcinogenicity probability prediction score for the compounds (≥0.94) when compared to ProTox-II’s carcinogenicity model carcinogenicity probability prediction score (≤0.51). Thus, the admetSAR’s non-carcinogenicity prediction for compounds **2** and **3** can be considered as the more accurate result (Tables S9–S11). Compounds **2** and **3** were predicted to be in the acute oral toxicity class III and be hepatotoxic, whereas **1** predicted to be in the acute oral toxicity class II and be non-hepatotoxic. The acute oral toxicity classes are categorized on the basis of the criteria from the United States Environmental Protection Agency (US EPA, https://www.epa.gov, accessed on 18 December 2021). The acute oral toxicity classes II and III for the admetSAR online prediction tool are equivalent to LD_50_ ranges of 50 mg/kg < LD_50_ ≤ 500 mg/kg and 500 mg/kg < LD_50_ ≤ 5000 mg/kg, respectively. The predicted hepatotoxicity and acute oral toxicity results did not correspond to the results that were obtained by ProTox-II. The acute toxicity class results did not correspond with results obtained in ProTox-II. ProTox-II predicted that compounds **2** and **3** were more orally toxic compared to **1**. ProTox-II’s hepatotoxicity model obtained higher non-hepatotoxicity probability prediction scores for **2** and **3** (0.73 and 0.72, respectively) when compared to admetSAR’s hepatotoxicity model hepatotoxic probability prediction scores (0.53 and 0.58, respectively). Thus, the ProTox-II non-hepatotoxicity prediction for compounds **2** and **3** can be considered to be the more accurate result. The compounds were predicted to be a substrate for the CYP450 enzyme CYP3A4. Compounds **1**–**3** were predicted to inhibit CYP450 enzymes CYP3A4, CYP3A4 and CYP2C9, CYP3A4, and CYP2C9 and CYP2C19, respectively. One of the most common mechanisms which can lead to drug–drug interactions is the inhibition of the CYP450 enzymes. Inhibition of the CYP450 enzymes can lead to an increase or reduction in therapeutic potency and other pharmacokinetic pathways of co-administered therapeutic agents used in polypharmacy treatment regimes [56]. The compounds exhibited sufficient pharmacokinetic properties to be considered as potential 11β-HSD1 inhibitors. The ADME results of both SwissADME and admetSAR correlated, and predicted that the compounds were orally bioavailable, had high gastrointestinal absorption, were not substrates to a majority of the CYP450 enzymes, and inhibited several CYP450 enzymes. The compounds were also predicted to permeate across the BBB. It has been previously hypothesized that the inhibition of 11β-HSD1 could treat cognitive impairment that is associated with early-stage Alzheimer’s Disease (AD) [56,57]. Therefore, the compound’s effects on the CNS and on CNS-related diseases should be considered. ProTox-II, STopTox and admetSAR all predicted that compounds **2** and **3** would be the most toxic of the three compounds, due to the addition of the pyridin-2-yl on the piperazine moiety (**2** and **3**), and the propene substitution on the triazole moiety (**3**).

## 3. Materials and Methods

### 3.1. Single-Crystal X-ray Diffraction

The X-ray intensity data of compounds **1** and **2** were measured at 160(1) K, using CuKα radiation (λ = 1.54184 Å) in a Rigaku OD XtaLAB Synergy, Dualflex, Pilatus 200K diffractometer from a micro-focus sealed X-ray tube and an Oxford liquid-nitrogen Cryostream cooler that was equipped with a Hybrid Pixel Array Detector. X-ray intensity data of compound **3** were measured under the same conditions of temperature (Oxford Instruments Cryojet XL cooler) and radiation (micro-focus X-ray source) in a Rigaku OD SuperNova/Atlas area-detector diffractometer that was equipped with a CCD plate detector. In all of the cases, suitable crystals were mounted using polybutene oil on a flexible loop that was fixed on a goniometer head, and immediately transferred to the diffractometer. The pre-experiment, data collection, data reduction and analytical absorption correction [58] were performed with the program suite *CrysAlisPro* [59]. Using *Olex2* [60], the structures were solved with the SHELXT [61] small molecule structure solution program, and then refined with the SHELXL2018/3 program package [62] using full-matrix least-squares minimization on F^2^. In compound **1**, the ethyl group (atoms C1 and C2) was disordered over two orientations, with a site-occupancy factor of 0.904(4) for the major component. In compound **2**, the terminal pyridine ring showed a disorder over two sets of positions, but only the pyridine N and its neighboring C atom (N2-C5 bond) were effectively disordered with site-occupancy factor 0.741(17) for the major component. In all of the structures, the methyl H atoms were constrained to an ideal geometry [C–H = 0.98 Å and *U*_iso_(H) = 1.5*U*_eq_(C)], but were allowed to rotate freely about the C–C bonds. All of the remaining H atoms were placed in idealized positions [C−H = 0.95–0.99 Å and *U*_iso_(H) = 1.2*U*_eq_(C)], and were constrained to ride on their parent atoms. *PLATON* software was used to check the results of the X-ray analyses, and to perform the structural and geometrical calculations [42]. Molecular and supramolecular graphics were carried out using Mercury software [63].

### 3.2. Hirshfeld Surface and Energy Frameworks Analysis

In order to quantify the contributions of different intermolecular interactions observed in these structures, and to understand the nature of these interactions, we carried out Hirshfeld surface analyses [64] using the *CrystalExplorer* program [65]. The Hirshfeld surfaces were mapped over *d*_norm_ distance, and this value was calculated from *d*_e_ and *d*_i_ pairs of values, identified as the external and internal distances of an atom to the Hirshfeld surface, respectively, which are normalized to the van der Waals (vdW) radii of the corresponding atoms. Contacts that were smaller than the sum of the vdW radii of the two atoms resulted in a negative value, highlighted on the surface in red. Contacts that were close to the limit of the vdW radii are shown in white, and those contacts that were greater than the sum of the vdW radii are highlighted on the surface in blue. When mapping *d*_e_ and *d*_i_ on this surface, these two values are associated, resulting in relations that are combined in intervals of 0.01 Å, providing the so-called two-dimensional (2D) fingerprint plots [66].

Pairwise interaction energies and the corresponding energy frameworks were calculated using the accurate and efficient CE-B3LYP model energies for intermolecular interactions, based on B3LYP/6-31G(d,p) quantum mechanical charge distribution for unperturbed monomers. In these calculations, the total interaction energy was partitioned as the sum of the electrostatic (E_ele_), polarization (E_pol_), dispersion (E_dis_), and exchange-repulsion (E_rep_) terms, based on the calculated molecular wavefunctions. In order to obtain accurate interaction energies, scale factors were used for electrostatic (1.057), polarization (0.740), dispersion (0.871) and repulsion (0.618) energy terms. These scale factors were determined by fitting to a large set of pairwise interaction energies that were calculated from a counterpoise-corrected B3LYP-D2/6-31G(d,p) level of approximation [67,68].

### 3.3. Molecular Docking Studies

#### 3.3.1. Preparation of Protein and Ligands

The holo X-ray structure of 11β-HSD1 was obtained from the protein data bank (PDB ID: *4C7J*) [69], and chain A was chosen for molecular docking because it contains the least number of outliers on the residue property plots, accurate binding interactions and an appropriate ligand model. The most appropriate ligand model was determined using the following parameters: goodness of fit percentage = 88% and real space correlation coefficient = 0.961. The protein structure and the docking studies were conducted using the Molecular Operating Environment (MOE) 2020 software suite [70], using the following protocol: the unselected protein chains and the respective co-crystallized ligand, solvent and co-factors were removed. Thereafter, the crystallographic water molecules that were further than 4.5 Å from the ligand were removed. Atoms that were further than 8 Å from the ligand were fixed, and the receptor residues were tethered with a constraint value of 0.25 Å. The tethering of the protein residue-heavy atoms within 8 Å of the ligand ensured that no artificial movements from the original coordinates could occur during energy minimization [71]. The proteins were structurally prepared and protonated through the utilization of the built-in MOE structure preparation, and Protonate3D software tools using their default parameters. Finally, partial charges were corrected, and energy minimization was conducted that utilized the following parameters: forcefield: MMFF94x, and solvation: Born and gradient: 0.01. Once the structures were optimized, the fixed and tethered constraints were removed for molecular docking. The docking algorithm, which was chosen for these experiments, was based on induced fit docking, in order to allow for flexible interactions of the test ligand with the protein active site side chains. Hence, the constraints were removed to ensure that the active site side chains were able to flex during induced fit docking. The prepared protein structures were saved in .moe file format. The ligands that were used for molecular docking were drawn using the ACD/ChemSketch package [72], and saved in mol2 file format. Protonation and energy minimization of the ligands were conducted, utilizing the following parameters: forcefield: MMFF94x, and solvation: Born and gradient: 0.0001.

#### 3.3.2. Molecular Docking of Compounds **1**–**3**

Self-docking was used to validate the docking protocol, as well as to determine the protein structure’s suitability to successfully dock the native ligand. The native ligand, **4YQ**, was docked using the following docking parameters: placement: triangle matcher, placement score algorithm: London dG, returned poses: 100, refinement: induced fit, iterations: 1000, refinement score algorithm: GBVI/WSA dG, and scored poses: 5. The free energy of binding of the ligand molecule was estimated using the force field-based scoring function (GBVI/WSA) with the implicit solvent model. The implicit solvent model, however, does have its limitations, as it under- or overestimates the strength of the solvation binding free energy of water-solvation hydrogen bonds, resulting in varying binding free energy scores. However, even though the scores can be influenced by the inclusion of crystallographic waters, previous studies have shown that the inclusion of these waters increases docking pose accuracy [50,73,74,75]. Furthermore, previous studies have shown that there is minimal statistical significance between binding affinity scores and experimentally determined ligand affinities to their respective targets [73]. Therefore, we considered the accuracy of the docked binding pose to be more important than the influenced docking scores caused by the crystallographic waters.

The successfulness of the docked ligands was determined using a root mean squared deviation (RMSD)-based criteria between the docked **4YQ** and the crystallographic **4YQ**. A RMSD value of <2 Å for both the top pose (lowest binding affinity score pose) and average RMSD across the top five docked poses, was used to validate the ability of the docking protocol to predict realistic binding conformations and interactions. The self-docked **4YQ** top pose obtained an RMSD of 1.65 Å, and an average RMSD over the top five poses of 1.33 Å. Therefore, the validated molecular docking protocol was employed in this study. Test compounds **1**–**3** were imported into a combined database, and were docked using the validated docking protocol. The best docked ligand conformation of each compound was selected using the following criteria: lowest binding affinity score within the top five binding conformations, and best interactions with important 11β-HSD1 active site residues. The best binding pose of each compound was visually inspected, and the interactions with the binding pocket residues were analyzed using the online servers Protein-Ligand Interaction Profiler (PLIP, https://plip-tool.biotec.tu-dresden.de, accessed on 10 December 2021) [76], Analysis of Protein-Ligand Interactions (nAPOLI, http://bioinfo.dcc.ufmg.br/napoli/, accessed on 10 December 2021) [77], Pymol molecular graphics system [78], and the MOE 2020 software suite [70]. The nAPOLI binding interaction analysis tool parameters were set to default, except for the hydrogen bond parameters: maximum donor atom to acceptor atom distance, and maximum donor to hydrogen distance were set to 4.1 Å and 3.5 Å, respectively. The nAPOLI hydrogen bond parameters were altered to correlate with the PLIP hydrogen bond parameters. The built-in scoring function of MOE, S-score, was used to predict the binding affinity (kcal/mol) of each ligand with the protein active site after docking.

### 3.4. *In Silico* Absorption, Distribution, Metabolism, Excretion and Toxicity (ADMET) Studies

The ProTox-II web-based toxicity prediction tool (https://tox-new.charite.de/protoxII, accessed on 15 January 2022) was employed to predict the toxic dose (mg/kg), toxicity class, toxicity targets and the pathway of the compounds [79]. The SwissADME online ADME tool was employed to predict the ADME properties of the compounds (http://www.swissadme.ch, accessed on 15 January 2022) [54]. The STopTox online toxicity prediction tool was employed to predict acute toxicities of the compounds in humans. The acute toxicity prediction assessments included acute oral, dermal and inhalation toxicity, as well as skin and eye irritation and corrosion, and skin sensitization (https://stoptox.mml.unc.edu, accessed on 15 January 2022) [51]. The AdmetSAR online ADMET tool was employed to further predict the ADMET properties of the compounds (http://lmmd.ecust.edu.cn/admetsar2/, accessed on 18 December 2021) [80,81].

## 4. Conclusions

Three adamantane-linked 1,2,4-triazole *N*-Mannich bases were synthesized, and these compounds were characterized using single-crystal X-ray diffraction. The substituent effect on the intermolecular interactions was investigated using Hirshfeld surface analysis. The results suggested that intermolecular H⋯H contacts were predominant in the solid-state structures. Despite the low contributions of H⋯N, H⋯O, H⋯S and H⋯C contacts towards crystal packing, these contacts played significant roles in stabilizing the crystal structures of compounds **1**–**3**. The energy frameworks for the crystal structures suggested that the dispersion forces were predominant in building the packing of molecules in the solid state. The pairwise interaction energy analysis indicated that the strength of dimers mediated by C–H⋯O and C–H⋯N interaction was comparable. We also noted that the stability of the molecular dimer formed by the C–H⋯π interaction was relatively higher compared to dimers that were formed by other interactions. Compounds **1**–**3** were docked within the 11β-HSD1 protein active site, co-crystallized with **4YQ**. The compounds exhibited hydrogen bond binding interactions with Leu217, which were found at the primary entrance pocket of the protein, as well as at key catalytic residues Ser170 and Tyr183. Compound **2** displayed the greatest potential as an 11β-HSD1 inhibitor, as it obtained a lower binding affinity score (−8.92 kcal/mol) compared to the potent co-crystallized 11β-HSD1 inhibitor **4YQ** (−8.48 kcal/mol). Overall, the compounds exhibited promising predicted pharmacokinetic properties, as they were predicted to be well absorbed, distributed and unlikely to be easily metabolized by CYP450 enzymes. Compounds **2** and **3** were predicted to be the most toxic out of the three compounds. The addition of the pyridin-2-yl substitution on the *N*-position of the piperazine moiety greatly increased predictability of the compound’s toxicity. Compounds **1** and **2** exhibited potential as 11β-HSD1 inhibitors, as they showed promising molecular docking binding affinity scores, interacted with important active site residues, as well as showed favorable ADME predictions. Compound **2** obtained the greatest binding affinity score and strong hydrogen bond interactions with important residues. However, compound **2** was predicted to be one of the most toxic compounds in this study. Therefore, optimization on the *N*-group substitution on the piperazine, i.e., bioisosteric replacement, should be considered to retain 11β-HSD1 inhibitory activity, while reducing the compound’s toxicity profile. Compound **1** obtained a similar binding affinity score to **4YQ**, and was predicted to be the least toxic. Further structural optimization of compound **1** on the *N*-group substitution of the piperazine moiety with a stronger electron withdrawing group is recommended, in order to improve its binding interactions with the primary entrance binding site residues, as well as to improve its binding affinity score, while retaining the compound’s low predicted toxicity profile.

## Figures and Tables

**Table 1:** Crystallographic data and structure refinement parameters of compounds **1**, **2** and **3**.

	Compound 1	Compound 2	Compound 3
Empirical formula	C_22_H_35_N_5_O_2_S	C_24_H_34_N_6_S	C_25_H_34_N_6_S
Formula weight	433.61	438.63	450.64
Temperature (K)	160 (1)	160 (1)	160 (1)
Crystal system	Monoclinic	Monoclinic	Monoclinic
Space group	*P*2_1_/*c*	*P*2_1_	*P*2_1_/*n*
*a, b, c* (Å)	13.73179 (19), 1.46565 (15), 14.2961 (2)	6.33861 (5), 13.40028 (10), 13.82724 (9)	11.10030 (10), 7.8744 (2), 23.7572 (2)
*α, β, γ* (°)	90, 100.5825 (15), 90	90, 102.1174 (7), 90	90, 96.8490 (10), 90
Volume/Å^3^	2212.54 (6)	1148.307 (14)	4680.06 (8)
*Z*	4	2	8
Radiation type	Cu *K*α (λ = 1.54184 Å)	Cu *K*α (λ = 1.54184 Å)	Cu *K*α (λ = 1.54184 Å)
Calculated density (g/cm^3^)	1.302	1.269	1.279
*µ* (mm^−1^)	1.527	1.426	1.415
Crystal size (mm^3^)	0.05 × 0.04 × 0.02	0.2 × 0.12 × 0.06	0.2 × 0.11 × 0.08
Diffractometer	Rigaku OD XtaLAB Synergy, Dualflex, Pilatus 200K		Rigaku OD SuperNova/Atlas area-detector
*T_min_, T_max_*	0.481, 0.731	0.831, 0.933	0.806, 0.926
2Θ range for data collection (°)	6.548–149	6.538–149	6.204–149.006
Index ranges	−17 ≤ h ≤ 17, −6 ≤ k ≤ 14, −17 ≤ l ≤ 17	−7 ≤ h ≤ 7, −16 ≤ k ≤ 16, −16 ≤ l ≤ 17	−13 ≤ h ≤ 13, −22 ≤ k ≤ 22, −27 ≤ l ≤ 29
Reflections collected	24,276	23,690	48,761
Independent reflections	4529 [*R*_int_ = 0.0333, *R*_sigma_ = 0.0257]	4681 [*R*_int_ = 0.0152, *R*_sigma_ = 0.0096]	9565 [*R*_int_ = 0.0308, *R*_sigma_ = 0.0199]
Data/restraints/parameters	4529/38/293	4681/65/301	9565/0/577
Goodness-of-fit on F^2^	1.050	1.049	1.034
Final *R* indexes [I ≥ 2σ (I)]	*R*_1_ = 0.0344, w*R*_2_ = 0.0878	*R*_1_ = 0.0255, w*R*_2_ = 0.0702	*R*_1_ = 0.0336, w*R*_2_ = 0.0864
Final *R* indexes [all data]	*R*_1_ = 0.0397, w*R*_2_ = 0.0912	*R*_1_ = 0.0259, w*R*_2_ = 0.0704	*R*_1_ = 0.0406, w*R*_2_ = 0.0908
Δρ_max_/Δρ_min_ (e Å^−3^)	0.26/−0.25	0.15/−0.16	0.22/−0.22
H-atom treatment	H-atom parameters constrained		
CCDC number	2,053,088	2,053,089	2,053,090

**Table 2:** Selected hydrogen-bond geometry (Å, °) for compounds **1**, **2** and **3**.

D-H⋯A	D-H	H⋯A	D⋯A	D-H⋯A
**Compound 1**				
C5-H5B‧‧‧O2^i^	0.99	2.40	3.2952(17)	150
C4-H4B⋯O1 (intramolecular)	0.99	2.31	2.7330(18)	105
C7-H7A⋯O2 (intramolecular)	0.99	2.37	2.7781(19)	104
**Compound 2**				
C1-H1A⋯N5^ii^	0.95	2.75	3.633(3)	155
C13-H13B⋯S1^iii^	0.99	3.19	3.845(3)	125
C21-H21A⋯* Cg1^iv^	0.99	2.72	3.683(3)	165
C23-H23A⋯Cg1^v^	0.99	2.74	3.610(3)	147
**Compound 3**				
C39-H39⋯N7^vi^	0.95	2.74	3.599(3)	151
C8-H8B⋯N11 between conformers	0.99	2.75	3.712(3)	163
C35-H35B″ïS1 between conformers	0.99	2.80	3.649(3)	144
C10-H10A⋯S2^vii^	0.99	2.76	3.642(3)	150
C50-H50A⋯Cg1^viii^	0.99	2.78	3.501(2)	130
C24-H24B⋯Cg1^ix^	0.99	2.72	3.537(2)	140

Symmetry codes: (i) x,1/2 − y,1/2 + z; (ii) 2 − x,−1/2 + y,1 − z; (iii) 1 − x,1/2 + y,2 − z; (iv) 1 − x,1/2 + y,1 − z; (v) x,1 + y, z; (vi) 3/2 − x,1/2 + y,3/2 − z; (vii) 1 + x,y,z; (viii) 1/2 − x,1/2 + y,3/2 − z; (ix) 1/2 − x,−1/2 + y,3/2 − z. * Cg1 is the centroid of the pyridine ring.

**Table 3:** Selected CrystalExplorer CE-B3LYP interaction energies (kJ/mol) for compounds **1**, **2** and **3**. N is the number of molecules with a molecular centroid-to-centroid distance R (Å). Electron density was calculated using B3LYP/6-31G(d,p) model energies. Symop is the symmetry operation. Note: scale factors used to determine E_tot_: E_ele_ = 1.057; E_pol_ = 0.740; E_dis_ = 0.871; E_rep_ = 0.618.

N	Symop	R	E_ele_	E_pol_	E_dis_	E_rep_	E_tot_
**Compound 1**							
2	x, −y + 1/2, z + 1/2 C5-H5B⋯O2^i^	7.25	−16.3	−8.4	−53.6	41.4	−44.5
1	−x, −y, −z pair of inversion related molecules	4.72	−36.9	−6.7	−151.9	128.0	−97.2
1	−x, −y, −z interaction between pairs of inversion related molecules	7.08	−15.2	−2.4	−57.8	35.8	−46.0
2	−x, y + 1/2, −z + 1/2	14.19	−6.3	−1.6	−27.0	23.2	−17.0
2	x, −y + 1/2, z + 1/2	14.32	−9.5	−1.7	−24.1	20.8	−19.5
2	x, y, z	13.73	−5.2	−0.8	−32.3	22.0	−20.6
2	−x, y + 1/2, −z + 1/2	8.59	−8.7	−2.0	−31.4	16.7	−27.7
2	−x, y + 1/2, −z + 1/2	9.90	−8.9	−2.8	−18.6	14.8	−18.5
1	−x, −y, −z	15.36	0.4	−0.2	−11.1	7.2	−5.0
**Compound 2**							
2	−x, y + 1/2, −z C1-H1A⋯N5^ii^ along [010]	9.97	−21.9	−5.6	−44.6	27.5	−49.1
2	x, y, z along [100]	6.34	−14.1	−9.9	−73.7	62.2	−48.0
2	−x, y + 1/2, −z C21-H21A⋯Cg1^iii^ along [010]	9.09	−27.4	−6.4	−69.1	31.3	−74.5
2	x, y, z	13.40	−11.1	−1.9	−37.4	14.6	−36.7
2	−x, y + 1/2, −z	11.23	−12.0	−4.6	−24.2	21.3	−24.0
2	−x, y + 1/2, −z	10.30	−6.5	−3.5	−33.3	22.0	−24.9
**Compound 3**							
1	Between conformers	6.56	−5.1	−1.2	−37.1	21.7	−25.2
1	Between conformers	6.66	−3.1	−0.9	−14.5	7.0	−12.3
2	−x + 1/2, y + 1/2, −z + 1/2	12.00	−3.1	−0.9	−14.5	7.0	−12.3
2	−x + 1/2, y + 1/2, −z + 1/2	10.24	−5.1	−1.2	−37.1	21.7	−25.2
1	Between conformers	11.93	−7.1	−4.6	−45.6	20.3	−38.1
1	−x, −y, −z	11.86	−12.6	−3.1	−10.3	6.6	−20.6

**Table 4:** Tabulated binding affinity scores (kcal/mol), hydrogen bond interactions, aromatic stacking interactions and water bridge interactions of **4YQ** and compounds **1**–**3** docked within *4C7J*.

Compounds	Binding Affinity Scores (kcal/mol)	Hydrogen Bond Interactions	Aromatic Stacking Interactions	Water Bridge Interactions
**4YQ**	−8.48	Thr124 ^b^ Ser170 ^c^ Tyr183 ^c^ Leu217 ^c^ Asp259 ^c^	None ^c^	Thr122 ^b^ Thr124 ^a^ Leu217 ^b^ Gln234 ^b^ Ala236 ^b^ Ser260 ^b^
**Compound 1**	−8.46	Ser170 ^a^ Tyr183 ^c^ Leu217 ^b^	Tyr183 ^b^	None ^c^
**Compound 2**	−8.92	Ser170 ^a^ Tyr183 ^c^ Leu217 ^c^	Tyr177 ^b^ Tyr183 ^b^	None ^c^
**Compound 3**	−7.50	Ser170 ^a^ Tyr183 ^c^ Leu217 ^c^	Tyr177 ^b^ Tyr183 ^b^	None ^c^

^a^ Represents interactions determined using PLIP online binding interaction analysis tools, ^b^ represents interactions determined using nAPOLI online binding interaction analysis tools, and ^c^ represents interactions determined using PLIP and nAPOLI online binding interaction analysis tools.

**Table 5:** *In silico* toxicity prediction assessment of various toxicity models for compounds **1**–**3** using online toxicity prediction tools ProTox-II, STopTox and admetSAR.

Compound 1						
Toxicity and Target Classification	ProTox-II	Probability	STopTox	Confidence	admetSAR	Probability
Oral LD_50_ (mg/kg) ^a^	1000	-	-	-	-	-
Oral Toxicity class	IV ^b^	-	-	-	II ^c^	0.45
Acute oral toxicity	-	-	Yes	78%	-	-
Hepatotoxicity	No	0.60	-	-	No	0.70
Carcinogenicity	No	0.55	-	-	No	0.90
Immunotoxicity	No	0.99	-	-	-	-
Mutagenicity	No	0.62	-	-	No	0.51
Cytotoxicity	No	0.73	-	-	-	-
Acute inhalation toxicity	-	-	No	54%	-	-
Acute dermal toxicity	-	-	No	64%	-	-
Eye irritation and corrosion	-	-	Yes	71%	No	>0.95
Skin irritation and corrosion	-	-	No	60%	-	-
Skin sensitization	-	-	No	60%	-	-
**Compound 2**						
Oral LD_50_ (mg/kg) ^a^	162	-	-	-	-	-
Oral Toxicity class	III ^b^	-	-	-	III ^c^	0.45
Acute oral toxicity	-	-	Yes	78%	-	-
Hepatotoxicity	No	0.73	-	-	Yes	0.53
Carcinogenicity	Yes	0.51	-	-	No	0.94
Immunotoxicity	No	0.95	-	-	-	-
Mutagenicity	No	0.59	-	-	No	0.52
Cytotoxicity	No	0.77	-	-	-	-
Acute inhalation toxicity	-	-	Yes	53%	-	-
Acute dermal toxicity	-	-	No	65%	-	-
Eye irritation and corrosion	-	-	Yes	54%	No	>0.97
Skin irritation and corrosion	-	-	Yes	50%	-	-
Skin sensitization	-	-	No	60%	-	-
**Compound 3**						
Oral LD_50_ (mg/kg) ^a^	162	-	-	-	-	-
Oral Toxicity class	III ^b^	-	-	-	III ^c^	0.52
Acute oral toxicity	-	-	Yes	65%	-	-
Hepatotoxicity	No	0.72	-	-	Yes	0.58
Carcinogenicity	Yes	0.50	-	-	No	0.94
Immunotoxicity	No	0.96	-	-	-	-
Mutagenicity	No	0.60	-	-	No	0.55
Cytotoxicity	No	0.77	-	-	-	-
Acute inhalation toxicity	-	-	Yes	55%	-	-
Acute dermal toxicity	-	-	No	66%	-	-
Eye irritation and corrosion	-	-	Yes	62%	No	>0.95
Skin irritation and corrosion	-	-	Yes	50%	-	-
Skin sensitization	-	-	Yes	60%	-	-

^a^ LD_50_ represents the dose required to kill 50% of the test subjects. ^b^ Oral toxicity classes III and IV are equivalent to LD_50_ ranges of 50 mg/kg < LD_50_ ≤ 300 mg/kg and 300 mg/kg < LD_50_ ≤ 2000 mg/kg, respectively. ^c^ Oral toxicity classes II and III are equivalent to LD_50_ ranges of 50 mg/kg < LD_50_ ≤ 500 mg/kg and 500 mg/kg < LD_50_ ≤ 5000 mg/kg, respectively.

**Table 6:** *In silico* pharmacokinetic prediction assessment of various ADME models of compounds **1**, **2** and **3**, using online ADME prediction tools SwissADME and admetSAR.

Compound 1		
Pharmacokinetic Target Classification	SwissADME	admetSAR
Human oral bioavailability	0.55	Yes
GI ^a^ absorption	High	Yes
Caco-2 permeability ^b^	-	No
BBB ^c^	Yes	Yes
P-gp ^d^ substrate	No	No
P-gp ^d^ inhibitor	-	No
CYP1A2 ^e^ inhibitor	No	No
CYP2C9 ^e^ inhibitor	Yes	No
CYP2C19 ^e^ inhibitor	Yes	No
CYP2D6 ^e^ inhibitor	Yes	No
CYP3A4 ^e^ inhibitor	Yes	Yes
CYP2C9 ^e^ Substrate	-	No
CYP2D6 ^e^ Substrate	-	No
CYP3A4 ^e^ Substrate	-	Yes
**Compound 2**		
Human oral bioavailability	0.55	Yes
GI ^a^ absorption	High	Yes
Caco-2 permeability ^b^	-	No
BBB ^c^	Yes	Yes
P-gp ^d^ substrate	No	Yes
P-gp ^d^ inhibitor	-	No
CYP1A2 ^e^ inhibitor	No	No
CYP2C9 ^e^ inhibitor	Yes	No
CYP2C19 ^e^ inhibitor	Yes	Yes
CYP2D6 ^e^ inhibitor	Yes	No
CYP3A4 ^e^ inhibitor	Yes	Yes
CYP2C9 ^e^ substrate	-	No
CYP2D6 ^e^ substrate	-	No
CYP3A4 ^e^ substrate	-	Yes
**Compound 3**		
Human oral bioavailability	0.55	Yes
GI ^a^ absorption	High	Yes
Caco-2 permeability ^b^	-	No
BBB ^c^	Yes	Yes
P-gp ^d^ substrate	No	No
P-gp ^d^ inhibitor	-	Yes
CYP1A2 ^e^ inhibitor	No	No
CYP2C9 ^e^ inhibitor	Yes	Yes
CYP2C19 ^e^ inhibitor	Yes	Yes
CYP2D6 ^e^ inhibitor	Yes	No
CYP3A4 ^e^ inhibitor	Yes	Yes
CYP2C9 ^e^ substrate	-	No
CYP2D6 ^e^ substrate	-	No
CYP3A4 ^e^ substrate	-	Yes

^a^ GI = gastrointestinal, ^b^ Caco-2 = human colorectal adenocarcinoma cell, ^c^ BBB = blood-brain barrier, ^d^ P-gp = P-glycoprotein, and ^e^ represents various subtypes of the CYP450 metabolizing enzyme.

## Data Availability

The crystal structures were resolved using SHELXT, and refined by the SHELXL2018/3 program package, both contained in the *Olex2* program (https://www.olexsys.org/). The evaluation of the geometric parameters in the molecular and supramolecular structures were performed using a combination of *PLATON* (http://www.platonsoft.nl/platon/) and *Mercury* (https://www.ccdc.cam.ac.uk/solutions/csd-core/components/mercury/) software. Hirshfeld surface, energy frameworks and HOMO-LUMO analyses of the crystallographic information were performed using the program *CrystalExplorer* (https://crystalexplorer.scb.uwa.edu.au/). The supplementary crystallographic data were obtained free of charge from the Cambridge Crystallographic Data Centre (www.ccdc.cam.ac.uk/data_request/cif). The compounds were CCDC-2053088 (compound **1**), CCDC-2053089 (compound **2**) and CCDC-2053090 (compound **3**). Further information and requests for resources used in the molecular docking analysis and *in silico* ADMET prediction analysis section should be directed to, and will be fulfilled by the corresponding author. All input and output data used and generated in the molecular docking analysis and *in silico* ADMET prediction analysis sections will be made available without restriction by the corresponding author. Software and webservers used: Molecular Operating Environment (https://www.chemcomp.com/), Pymol2.5 (https://github.com/schrodinger/pymol-open-source), Protein-Ligand Interaction Profiler (PLIP, https://plip-tool.biotec.tu-dresden.de/plip-web/plip), Analysis of Protein-Ligand Interactions (nAPOLI, http://bioinfo.dcc.ufmg.br/napoli/), ProTox-II (https://tox-new.charite.de/protox_II/), SwissADME (http://www.swissadme.ch/), STopTox (https://stoptox.mml.unc.edu/) and AdmetSAR2 (http://lmmd.ecust.edu.cn/admetsar2).

## Supplementary Materials

The following are available online at: http://https://www.mdpi.com/article/10.3390/molecules27217403/s1. Figure S1: Visual representations of binding interactions of compound **4YQ** within the *4C7J* active site using PLIP and Pymol molecular graphics system; Figure S2: Visual representations of binding interactions of compound **1** within the *4C7J* active site using PLIP and Pymol molecular graphics system; Figure S3: Visual representations of binding interactions of compound **2** within the *4C7J* active site using PLIP and Pymol molecular graphics system; Figure S4: Visual representations of binding interactions of compound **3** within the *4C7J* active site using PLIP and Pymol molecular graphics system; Figure S5: Visual representation of the predicted toxicity results of compound **2** obtained from the online toxicity prediction tool ProTox-II; Figure S6: Visual representation of the predicted toxicity results of compound **2** obtained from the online toxicity prediction tool ProTox-II; Figure S7: Visual representation of the predicted toxicity results of compound **3** obtained from the online toxicity prediction tool ProTox-II; Figure S8: Visual representation of the predicted ADME results of compound **2** obtained from the online ADME prediction tool SwissADME; Figure S9: Visual representation of the predicted ADME results of compound **2** obtained from the online ADME prediction tool SwissADME; Figure S10: Visual representation of the predicted ADME results of compound **3** obtained from the online ADME prediction tool SwissADME; Figure S11. Visual representation of the predicted toxicity results of compound **1** obtained from the online toxicity prediction tool STopTox; Figure S12: Visual representation of the predicted toxicity results of compound **2** obtained from the online toxicity prediction tool STopTox; Figure S13: Visual representation of the predicted toxicity results of compound **3** obtained from the online toxicity prediction tool STopTox. Table S1: Tabulated binding interactions between **4YQ** and *4C7J* active site residues identified by PLIP binding interaction analysis tools; Table S2: Tabulated binding interactions between **4YQ** and *4C7J* active site residues identified by nAPOLI binding interaction analysis tools; Table S3: Tabulated binding interactions between **1** and *4C7J* active site residues identified by PLIP binding interaction analysis tools; Table S4: Tabulated binding interactions between **1** and *4C7J* active site residues identified by nAPOLI binding interaction analysis tools; Table S5: Tabulated binding interactions between **2** and *4C7J* active site residues identified by PLIP binding interaction analysis tools; Table S6: Tabulated binding interactions between **2** and *4C7J* active site residues identified by nAPOLI binding interaction analysis tools; Table S7: Tabulated binding interactions between **3** and *4C7J* active site residues identified by PLIP binding interaction analysis tools; Table S8: Tabulated binding interactions between **3** and *4C7J* active site residues identified by nAPOLI binding interaction analysis tools; Table S9: Tabulated toxicity prediction results of compounds **2** obtained from the web-based prediction tool admetSAR; Table S10: Tabulated toxicity prediction results of compounds **2** obtained from the web-based prediction tool admetSAR; Table S11: Tabulated toxicity prediction results of compounds **3** obtained from the web-based prediction tool admetSAR.

## Sample Availability

Samples of compounds **1**, **2** and **3** are available from the authors.
