# Supplementary material for: Weak Noncovalent Interactions in Three Closely Related Adamantane-Linked 1,2,4-Triazole N-Mannich Bases: Insights from Energy Frameworks, Hirshfeld Surface Analysis, In Silico 11β-HSD1 Molecular Docking and ADMET Prediction
Source: Molecules. 2022 Oct 31;27(21):7403. doi: 10.3390/molecules27217403 (PMC9658560; doi:10.3390/molecules27217403)
Supplement: Supplementary file 1 [file molecules-27-07403-s001.zip › molecules-1960649-supplementary.pdf]

Weak Noncovalent Interactions in Three Closely Related Adamantane-linked 1,2,4-triazole *N*-Mannich Bases: Insights from Energy Frameworks, Hirshfeld Surface Analysis, In-Silico 11β-HSD1 Molecular Docking and ADMET Prediction

Supporting Information

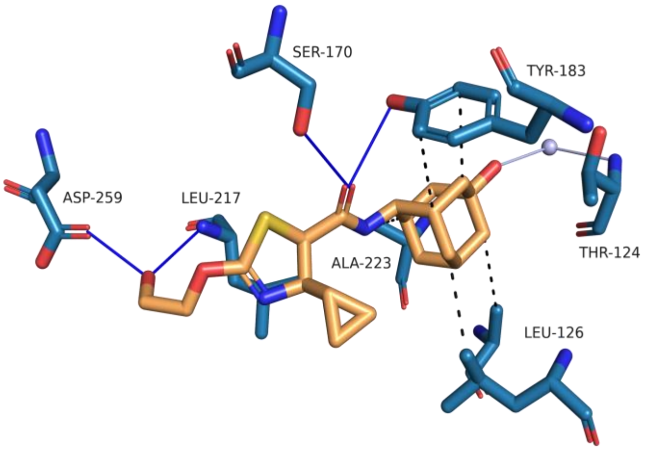

Figure S1. Visual representations of binding interactions of compound **4YQ** within *4C7J* active site using PLIP and Pymol molecular graphics system.

Table S1. Tabulated binding interactions between **4YQ** and *4C7J* active site residues identified by PLIP binding interaction analysis tools.

Residue	Amino Acid	Distance (Å)	Type of Interaction
126	LEU	3.56	Hydrophobic
183	TYR	3.68	Hydrophobic
183	TYR	3.91	Hydrophobic
223	ALA	3.71	Hydrophobic
226	ALA	3.87	Hydrophobic
170	SER	2.06	Hydrogen
183	TYR	2.30	Hydrogen
217	LEU	1.91	Hydrogen
259	ASP	1.65	Hydrogen
124	THR	2.74	Water bridge

Table S2. Tabulated binding interactions between **4YQ** and *4C7J* active site residues identified by nAPOLI binding interaction analysis tools.

Residue	Amino Acid	Distance (Å)
121	ILE	Hydrophobic
126	LEU	Hydrophobic
180	VAL	Hydrophobic
183	TYR	Hydrophobic
217	LEU	Hydrophobic
223	ALA	Hydrophobic
226	ALA	Hydrophobic
227	VAL	Hydrophobic
231	VAL	Hydrophobic
233	MET	Hydrophobic
124	THR	Hydrogen bond
170	SER	Hydrogen bond
183	TYR	Hydrogen bond
217	LEU	Hydrogen bond
259	ASP	Hydrogen bond
122	THR	Water bridge
217	LEU	Water bridge
234	GLN	Water bridge
236	ALA	Water bridge
260	SER	Water bridge

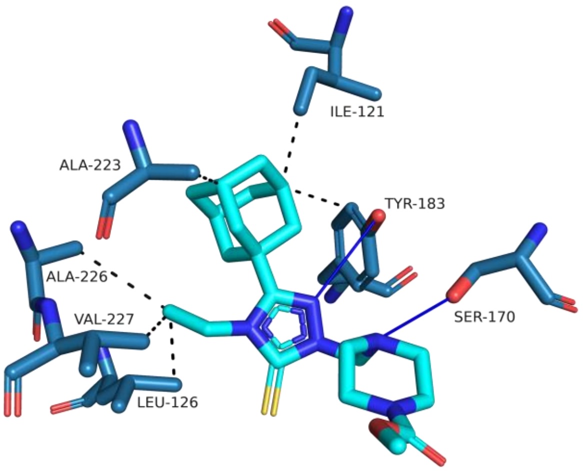

Figure S2. Visual representations of binding interactions of compound **1** within *4C7J* active site using PLIP and Pymol molecular graphics system.

Table S3. Tabulated binding interactions between **1** and *4C7J* active site residues identified by PLIP binding interaction analysis tools.

Residue	Amino Acid	Distance (Å)	Type of interaction
121	ILE	3.39	Hydrophobic
126	LEU	3.08	Hydrophobic
183	TYR	3.53	Hydrophobic
223	ALA	3.55	Hydrophobic
226	ALA	3.70	Hydrophobic
227	VAL	3.56	Hydrophobic
170	SER	3.11	Hydrogen
183	TYR	3.19	Hydrogen

Table S4. Tabulated binding interactions between **1** and *4C7J* active site residues identified by nAPOLI binding interaction analysis tools.

Residue	Amino Acid	Type of interaction
121	ILE	Hydrophobic
124	THR	Hydrophobic
126	LEU	Hydrophobic
180	VAL	Hydrophobic
183	TYR	Hydrophobic
222	THR	Hydrophobic
223	ALA	Hydrophobic
226	ALA	Hydrophobic
227	VAL	Hydrophobic
183	TYR	Aromatic stacking
183	TYR	Hydrogen bond
217	LEU	Hydrogen bond

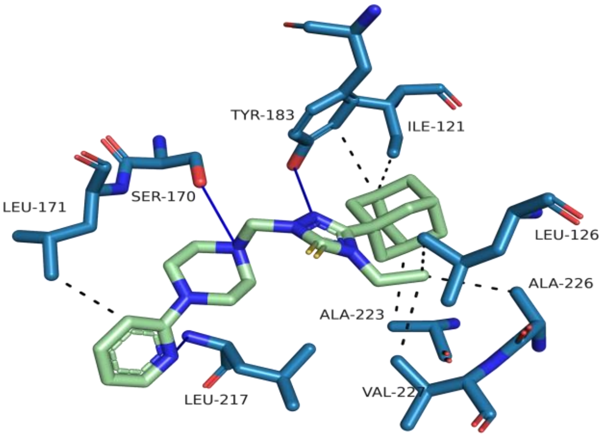

Figure S3. Visual representations of binding interactions of compound **2** within *4C7J* active site using PLIP and Pymol molecular graphics system.

Table S5. Tabulated binding interactions between **2** and *4C7J* active site residues identified by PLIP binding interaction analysis tools.

Residue	Amino Acid	Distance (Å)	Type of interaction
121	ILE	3.39	Hydrophobic
126	LEU	3.08	Hydrophobic
171	LEU	3.57	Hydrophobic
183	TYR	3.52	Hydrophobic
223	ALA	3.55	Hydrophobic
226	ALA	3.72	Hydrophobic
227	VAL	3.53	Hydrophobic
170	SER	3.09	Hydrogen
183	TYR	3.21	Hydrogen
217	LEU	2.21	Hydrogen

Table S6. Tabulated binding interactions between **2** and *4C7J* active site residues identified by nAPOLI binding interaction analysis tools.

Residue	Amino Acid	Type of interaction
121	ILE	Hydrophobic
124	THR	Hydrophobic
126	LEU	Hydrophobic
171	LEU	Hydrophobic
177	TYR	Hydrophobic
180	VAL	Hydrophobic
183	TYR	Hydrophobic
222	THR	Hydrophobic
223	ALA	Hydrophobic
226	ALA	Hydrophobic
227	VAL	Hydrophobic
233	MET	Hydrophobic
264	THR	Hydrophobic
177	TYR	Aromatic stacking
183	TYR	Aromatic stacking
183	TYR	Hydrogen bond
217	LEU	Hydrogen bond

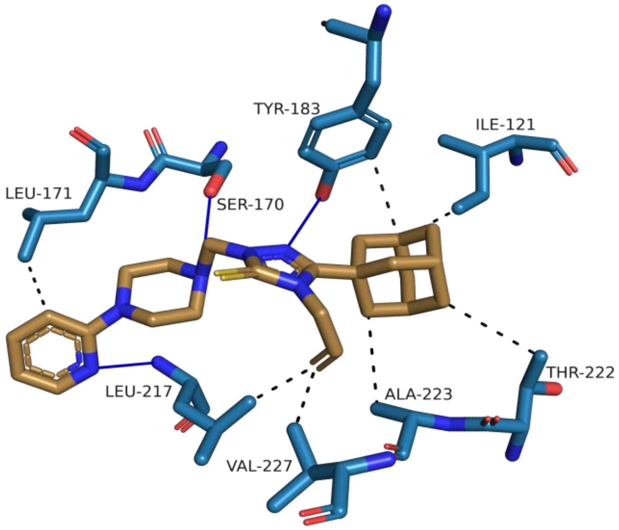

Figure S4. Visual representations of binding interactions of compound **3** within *4C7J* active site using PLIP and Pymol molecular graphics system.

Table S7. Tabulated binding interactions between **3** and *4C7J* active site residues identified by PLIP binding interaction analysis tools.

Residue	Amino Acid	Distance (Å)	Type of interaction
121A	ILE	3.17	Hydrophobic
171A	LEU	3.59	Hydrophobic
183A	TYR	3.31	Hydrophobic
217A	LEU	2.96	Hydrophobic
222A	THR	3.99	Hydrophobic
223A	ALA	3.65	Hydrophobic
227A	VAL	2.86	Hydrophobic
170A	SER	3.34	Hydrogen
183A	TYR	3.09	Hydrogen
217A	LEU	2.14	Hydrogen

Table S8. Tabulated binding interactions between **3** and *4C7J* active site residues identified by nAPOLI binding interaction analysis tools.

Residue	Amino Acid	Type of interaction
121	ILE	Hydrophobic
124	THR	Hydrophobic
126	LEU	Hydrophobic
171	LEU	Hydrophobic
177	TYR	Hydrophobic
183	TYR	Hydrophobic
217	LEU	Hydrophobic
222	THR	Hydrophobic
223	ALA	Hydrophobic
226	ALA	Hydrophobic
227	VAL	Hydrophobic
233	MET	Hydrophobic
264	THR	Hydrophobic
177	TYR	Aromatic stacking
183	TYR	Aromatic stacking
183	TYR	Hydrogen bond
217	LEU	Hydrogen bond

Figure S5. Visual representation of the predicted toxicity results of compound **2** obtained from the online toxicity prediction tool ProTox-II.

Oral toxicity prediction results for input compound

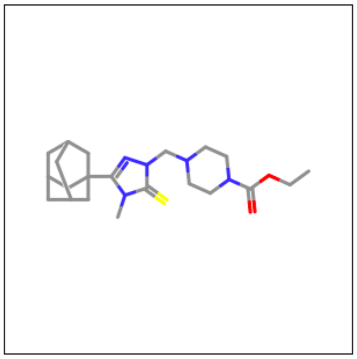

Predicted LD50: 1000mg/kg

Predicted Toxicity Class: 4

1 2 3 4 5 6

Average similarity: 41.07%

Prediction accuracy: 54.26%

20% 40% 60% 80%

Name	User defined
Molweight	419.58
Number of hydrogen bond acceptors	39
Number of hydrogen bond donors	0
Number of atoms	62
Number of bonds	66
Number of rotable bonds	6
Molecular refractivity	122.18
Topological Polar Surface Area	87.62
octanol/water partition coefficient(logP)	3.03

Toxicity Model Report

Classification	Target	Shorthand	Prediction	Probability
Organ toxicity	Hepatotoxicity	dili	Inactive	0.60
Toxicity end points	Carcinogenicity	carcino	Inactive	0.55
Toxicity end points	Immunotoxicity	immuno	Inactive	0.99
Toxicity end points	Mutagenicity	mutagen	Inactive	0.62
Toxicity end points	Cytotoxicity	cyto	Inactive	0.73
Tox21-Nuclear receptor signalling pathways	Aryl hydrocarbon Receptor (AhR)	nr_ahr	Inactive	0.80
Tox21-Nuclear receptor signalling pathways	Androgen Receptor (AR)	nr_ar	Inactive	0.97
Tox21-Nuclear receptor signalling pathways	Androgen Receptor Ligand Binding Domain (AR-LBD)	nr_ar_lbd	Inactive	0.95
Tox21-Nuclear receptor signalling pathways	Aromatase	nr_aromatase	Inactive	0.87
Tox21-Nuclear receptor signalling pathways	Estrogen Receptor Alpha (ER)	nr_er	Inactive	0.88
Tox21-Nuclear receptor signalling pathways	Estrogen Receptor Ligand Binding Domain (ER-LBD)	nr_er_lbd	Inactive	0.92
Tox21-Nuclear receptor signalling pathways	Peroxisome Proliferator Activated Receptor Gamma (PPAR-Gamma)	nr_ppar_gamma	Inactive	0.90
Tox21-Stress response pathways	Nuclear factor (erythroid-derived 2)-like 2/antioxidant responsive element (nrf2/ARE)	sr_are	Inactive	0.91
Tox21-Stress response pathways	Heat shock factor response element (HSE)	sr_hse	Inactive	0.91
Tox21-Stress response pathways	Mitochondrial Membrane Potential (MMP)	sr_mmp	Inactive	0.80
Tox21-Stress response pathways	Phosphoprotein (Tumor Supressor) p53	sr_p53	Inactive	0.87
Tox21-Stress response pathways	ATPase family AAA domain-containing protein 5 (ATAD5)	sr_atad5	Inactive	0.96

Oral toxicity class IV is equivalent to LD_{50} range of 300 mg/kg < LD_{50} ≤ 2000 mg/kg.

Figure S6. Visual representation of the predicted toxicity results of compound **2** obtained from the online toxicity prediction tool ProTox-II.

Oral toxicity prediction results for input compound

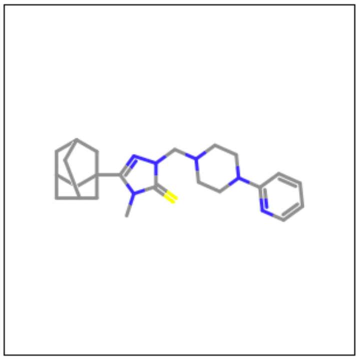

Predicted LD50: 162mg/kg

Predicted Toxicity Class: 3

1	2	3	4	5	6

Average similarity: 40.29%

Prediction accuracy: 54.26%

20% 40% 60% 80%

Name	User defined
Molweight	424.61
Number of hydrogen bond acceptors	37
Number of hydrogen bond donors	0
Number of atoms	62
Number of bonds	67
Number of rotable bonds	4
Molecular refractivity	128.92
Topological Polar Surface Area	74.21
octanol/water partition coefficient(logP)	3.6

Toxicity Model Report

Classification	Target	Shorthand	Prediction	Probability
Organ toxicity	Hepatotoxicity	dili	Inactive	0.73
Toxicity end points	Carcinogenicity	carcino	Active	0.51
Toxicity end points	Immunotoxicity	immuno	Inactive	0.95
Toxicity end points	Mutagenicity	mutagen	Inactive	0.59
Toxicity end points	Cytotoxicity	cyto	Inactive	0.77
Tox21-Nuclear receptor signalling pathways	Aryl hydrocarbon Receptor (AhR)	nr_ahr	Inactive	0.80
Tox21-Nuclear receptor signalling pathways	Androgen Receptor (AR)	nr_ar	Inactive	0.98
Tox21-Nuclear receptor signalling pathways	Androgen Receptor Ligand Binding Domain (AR-LBD)	nr_ar_lbd	Inactive	0.97
Tox21-Nuclear receptor signalling pathways	Aromatase	nr_aromatase	Inactive	0.83
Tox21-Nuclear receptor signalling pathways	Estrogen Receptor Alpha (ER)	nr_er	Inactive	0.66
Tox21-Nuclear receptor signalling pathways	Estrogen Receptor Ligand Binding Domain (ER-LBD)	nr_er_lbd	Inactive	0.82
Tox21-Nuclear receptor signalling pathways	Peroxisome Proliferator Activated Receptor Gamma (PPAR-Gamma)	nr_ppar_gamma	Inactive	0.88
Tox21-Stress response pathways	Nuclear factor (erythroid-derived 2)-like 2/antioxidant responsive element (nrf2/ARE)	sr_are	Inactive	0.89
Tox21-Stress response pathways	Heat shock factor response element (HSE)	sr_hse	Inactive	0.89
Tox21-Stress response pathways	Mitochondrial Membrane Potential (MMP)	sr_mmp	Inactive	0.82
Tox21-Stress response pathways	Phosphoprotein (Tumor Supressor) p53	sr_p53	Inactive	0.87
Tox21-Stress response pathways	ATPase family AAA domain-containing protein 5 (ATAD5)	sr_atad5	Inactive	0.96

Oral toxicity class III is equivalent to LD_{50} range of 50 mg/kg < LD_{50} ≤ 300 mg/kg.

Figure S7. Visual representation of the predicted toxicity results of compound **3** obtained from the online toxicity prediction tool ProTox-II.

Oral toxicity prediction results for input compound

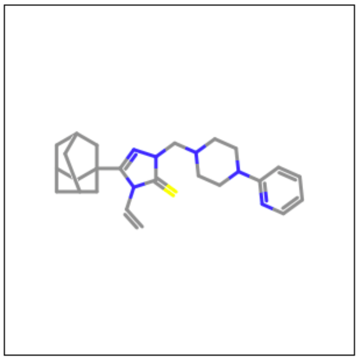

Predicted LD50: 162mg/kg

Predicted Toxicity Class: 3

1 2 3 4 5 6

Average similarity: 38.16%

Prediction accuracy: 23%

20% 40% 60% 80%

Name	User defined
Molweight	436.62
Number of hydrogen bond acceptors	37
Number of hydrogen bond donors	0
Number of atoms	63
Number of bonds	68
Number of rotable bonds	5
Molecular refractivity	134.05
Topological Polar Surface Area	74.21
octanol/water partition coefficient(logP)	4.16

Classification	Target	Shorthand	Prediction	Probability
Organ toxicity	Hepatotoxicity	dili	Inactive	0.72
Toxicity end points	Carcinogenicity	carcino	Active	0.50
Toxicity end points	Immunotoxicity	immuno	Inactive	0.96
Toxicity end points	Mutagenicity	mutagen	Inactive	0.60
Toxicity end points	Cytotoxicity	cyto	Inactive	0.77
Tox21-Nuclear receptor signalling pathways	Aryl hydrocarbon Receptor (AhR)	nr_ahr	Inactive	0.77
Tox21-Nuclear receptor signalling pathways	Androgen Receptor (AR)	nr_ar	Inactive	0.98
Tox21-Nuclear receptor signalling pathways	Androgen Receptor Ligand Binding Domain (AR-LBD)	nr_ar_lbd	Inactive	0.96
Tox21-Nuclear receptor signalling pathways	Aromatase	nr_aromatase	Inactive	0.81
Tox21-Nuclear receptor signalling pathways	Estrogen Receptor Alpha (ER)	nr_er	Inactive	0.69
Tox21-Nuclear receptor signalling pathways	Estrogen Receptor Ligand Binding Domain (ER-LBD)	nr_er_lbd	Inactive	0.84
Tox21-Nuclear receptor signalling pathways	Peroxisome Proliferator Activated Receptor Gamma (PPAR-Gamma)	nr_ppar_gamma	Inactive	0.87
Tox21-Stress response pathways	Nuclear factor (erythroid-derived 2)-like 2/antioxidant responsive element (nrf2/ARE)	sr_are	Inactive	0.86
Tox21-Stress response pathways	Heat shock factor response element (HSE)	sr_hse	Inactive	0.86
Tox21-Stress response pathways	Mitochondrial Membrane Potential (MMP)	sr_mmp	Inactive	0.80
Tox21-Stress response pathways	Phosphoprotein (Tumor Supressor) p53	sr_p53	Inactive	0.84
Tox21-Stress response pathways	ATPase family AAA domain-containing protein 5 (ATAD5)	sr_atad5	Inactive	0.94

Oral toxicity class III is equivalent to LD_{50} range of 50 mg/kg < LD_{50} ≤ 300 mg/kg.

Figure S8. Visual representation of the predicted ADME results of compound **2** obtained from the online ADME prediction tool SwissADME.

Molecule 1

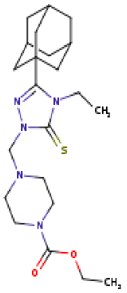

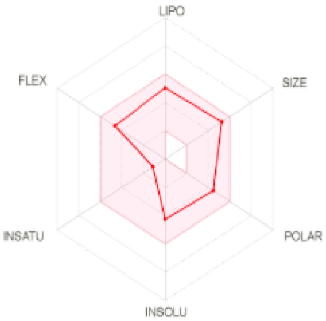

SMILES CCn1c(nn(c1=S)CN1CCN(CC1)C(=O)OCC)C12CC3CC(C2)CC(C1)C3

Physicochemical Properties	
Formula	C22H35N5O2S
Molecular weight	433.61 g/mol
Num. heavy atoms	30
Num. arom. heavy atoms	5
Fraction Csp3	0.86
Num. rotatable bonds	7
Num. H-bond acceptors	4
Num. H-bond donors	0
Molar Refractivity	126.98
TPSA	87.62 Å²

Lipophilicity	
Log $P_{o/w}$ (iLOGP)	4.52
Log $P_{o/w}$ (XLOGP3)	3.28
Log $P_{o/w}$ (WLOGP)	2.72
Log $P_{o/w}$ (MLOGP)	3.11
Log $P_{o/w}$ (SILICOS-IT)	2.68
Consensus Log $P_{o/w}$	3.26

Water Solubility	
Log *S* (ESOL)	-4.26
Solubility	2.40e-02 mg/ml ; 5.54e-05 mol/l
Class	Moderately soluble
Log *S* (Ali)	-4.79
Solubility	6.95e-03 mg/ml ; 1.60e-05 mol/l
Class	Moderately soluble
Log *S* (SILICOS-IT)	-2.74
Solubility	7.89e-01 mg/ml ; 1.82e-03 mol/l
Class	Soluble

Pharmacokinetics	
GI absorption	High
BBB permeant	No
P-gp substrate	No
CYP1A2 inhibitor	No
CYP2C19 inhibitor	Yes
CYP2C9 inhibitor	Yes
CYP2D6 inhibitor	Yes
CYP3A4 inhibitor	Yes
Log K_p (skin permeation)	-6.62 cm/s

Druglikeness	
Lipinski	Yes; 0 violation
Ghose	Yes
Veber	Yes
Egan	Yes
Muegge	Yes
Bioavailability Score	0.55

Medicinal Chemistry	
PAINS	0 alert
Brenk	1 alert: thiocarbonyl_group
Leadlikeness	No; 1 violation: MW>350
Synthetic accessibility	6.29

Figure S9. Visual representation of the predicted ADME results of compound **2** obtained from the online ADME prediction tool SwissADME.

Molecule 2

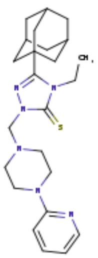

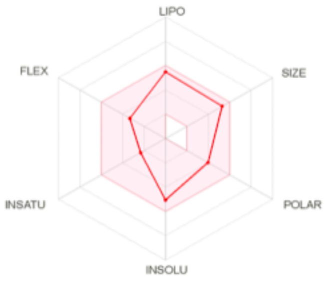

SMILES CCn1c(=S)n(nc1C12CC3CC(C2)CC(C1)C3)CN1CCN(CC1)c1ccccn1

Physicochemical Properties	
Formula	C24H34N6S
Molecular weight	438.63 g/mol
Num. heavy atoms	31
Num. arom. heavy atoms	11
Fraction Csp3	0.71
Num. rotatable bonds	5
Num. H-bond acceptors	3
Num. H-bond donors	0
Molar Refractivity	133.73
TPSA	74.21 Å²

Lipophilicity	
Log $P_{o/w}$ (iLOGP)	4.01
Log $P_{o/w}$ (XLOGP3)	4.10
Log $P_{o/w}$ (WLOGP)	3.16
Log $P_{o/w}$ (MLOGP)	3.22
Log $P_{o/w}$ (SILICOS-IT)	3.28
Consensus Log $P_{o/w}$	3.55

Water Solubility	
Log *S* (ESOL)	-5.08
Solubility	3.69e-03 mg/ml ; 8.41e-06 mol/l
Class	Moderately soluble
Log *S* (Ali)	-5.36
Solubility	1.90e-03 mg/ml ; 4.32e-06 mol/l
Class	Moderately soluble
Log *S* (SILICOS-IT)	-4.40
Solubility	1.75e-02 mg/ml ; 4.00e-05 mol/l
Class	Moderately soluble

Pharmacokinetics	
GI absorption	High
BBB permeant	Yes
P-gp substrate	No
CYP1A2 inhibitor	No
CYP2C19 inhibitor	Yes
CYP2C9 inhibitor	Yes
CYP2D6 inhibitor	Yes
CYP3A4 inhibitor	Yes
Log K_p (skin permeation)	-6.06 cm/s

Druglikeness	
Lipinski	Yes; 0 violation
Ghose	No; 1 violation: MR>130
Veber	Yes
Egan	Yes
Muegge	Yes
Bioavailability Score	0.55

Medicinal Chemistry	
PAINS	0 alert
Brenk	1 alert: thiocarbonyl_group
Leadlikeness	No; 2 violations: MW>350, XLOGP3>3.5
Synthetic accessibility	6.23

Figure S10. Visual representation of the predicted ADME results of compound **3** obtained from the online ADME prediction tool SwissADME.

Molecule 3

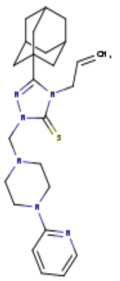

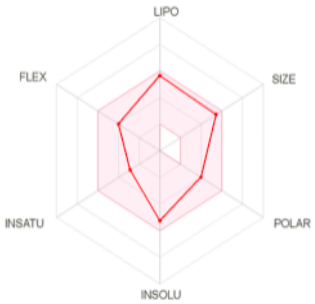

SMILES C=CCn1c(=S)n(nc1C12CC3CC(C2)CC(C1)C3)CN1CCN(CC1)c1ccc cn1

Physicochemical Properties	
Formula	C25H34N6S
Molecular weight	450.64 g/mol
Num. heavy atoms	32
Num. arom. heavy atoms	11
Fraction Csp3	0.64
Num. rotatable bonds	6
Num. H-bond acceptors	3
Num. H-bond donors	0
Molar Refractivity	138.06
TPSA	74.21 Å²

Lipophilicity	
Log $P_{o/w}$ (iLOGP)	4.23
Log $P_{o/w}$ (XLOGP3)	4.37
Log $P_{o/w}$ (WLOGP)	3.33
Log $P_{o/w}$ (MLOGP)	3.35
Log $P_{o/w}$ (SILICOS-IT)	3.66
Consensus Log $P_{o/w}$	3.79

Water Solubility	
Log *S* (ESOL)	-5.25
Solubility	2.56e-03 mg/ml ; 5.68e-06 mol/l
Class	Moderately soluble
Log *S* (Ali)	-5.64
Solubility	1.02e-03 mg/ml ; 2.27e-06 mol/l
Class	Moderately soluble
Log *S* (SILICOS-IT)	-4.45
Solubility	1.62e-02 mg/ml ; 3.59e-05 mol/l
Class	Moderately soluble

Pharmacokinetics	
GI absorption	High
BBB permeant	Yes
P-gp substrate	No
CYP1A2 inhibitor	No
CYP2C19 inhibitor	Yes
CYP2C9 inhibitor	Yes
CYP2D6 inhibitor	Yes
CYP3A4 inhibitor	Yes
Log K_p (skin permeation)	-5.95 cm/s

Druglikeness	
Lipinski	Yes; 0 violation
Ghose	No; 1 violation: MR>130
Veber	Yes
Egan	Yes
Muegge	Yes
Bioavailability Score	0.55

Medicinal Chemistry	
PAINS	0 alert
Brenk	2 alerts: isolated_alkene, thiocarbonyl_group
Leadlikeness	No; 2 violations: MW>350, XLOGP3>3.5
Synthetic accessibility	6.35

Table S9. Tabulated toxicity prediction results of compounds **1** obtained from the web-based prediction tool admetSAR.

ADMET predicted profile classification	Prediction	Probability
Human Intestinal Absorption	+	0.9873
Caco-2	-	0.5481
Blood Brain Barrier	+	0.9777
Human oral bioavailability	+	0.5143
Subcellular localzation	Mitochondria	0.6138
OATP2B1 inhibitior	-	0.8554
OATP1B1 inhibitior	+	0.9348
OATP1B3 inhibitior	+	0.9363
MATE1 inhibitior	-	0.7800
OCT2 inhibitior	-	0.6500
BSEP inhibitior	+	0.6672
P-glycoprotein inhibitior	-	0.6387
P-glycoprotein substrate	-	0.6459
CYP3A4 substrate	+	0.6616
CYP2C9 substrate	-	1.0000
CYP2D6 substrate	-	0.8477
CYP3A4 inhibition	+	0.5826
CYP2C9 inhibition	-	0.5311
CYP2C19 inhibition	-	0.5000
CYP2D6 inhibition	-	0.8358
CYP1A2 inhibition	-	0.6717
CYP inhibitory promiscuity	+	0.8338
UGT catelyzed	-	0.0000
Carcinogenicity (binary)	-	0.9000
Carcinogenicity (trinary)	Non-required	0.5954
Eye corrosion	-	0.9822
Eye irritation	-	0.9547
Ames mutagenesis	-	0.5100
Human ether-a-go-go inhibition	-	0.5792
micronuclear	+	0.6100
Hepatotoxicity	-	0.7000
Acute Oral Toxicity (c)	II	0.4543
Estrogen receptor binding	+	0.7321
Androgen receptor binding	-	0.6066
Thyroid receptor binding	+	0.6372
Glucocorticoid receptor binding	-	0.4856
Aromatase binding	+	0.5511
PPAR gamma	-	0.5113

+ = positive to specified ADMET classification, - = negative to specified ADMET classification, Acute Oral Toxicity catergory II = median lethal dose of compound ranges between 50 mg/kg < LD_{50} < 500 mg/kg.

Table S10. Tabulated toxicity prediction results of compounds **2** obtained from the web-based prediction tool admetSAR.

ADMET predicted profile classification	Prediction	Probability
Human Intestinal Absorption	+	0.9848
Caco-2	-	0.7061
Blood Brain Barrier	+	0.9844
Human oral bioavailability	+	0.6429
Subcellular localization	Mitochondria	0.3950
OATP2B1 inhibitor	-	1.0000
OATP1B1 inhibitor	+	0.9379
OATP1B3 inhibitor	+	0.9388
MATE1 inhibitor	-	0.8600
OCT2 inhibitor	-	0.6500
BSEP inhibitor	+	0.8420
P-glycoprotein inhibitor	-	0.4617
P-glycoprotein substrate	+	0.5558
CYP3A4 substrate	+	0.6589
CYP2C9 substrate	-	1.0000
CYP2D6 substrate	-	0.8369
CYP3A4 inhibition	+	0.8342
CYP2C9 inhibition	-	0.6842
CYP2C19 inhibition	+	0.6418
CYP2D6 inhibition	-	0.7999
CYP1A2 inhibition	-	0.5000
CYP inhibitory promiscuity	+	0.9202
UGT catalyzed	-	0.0000
Carcinogenicity (binary)	-	0.9429
Carcinogenicity (trinary)	Non-required	0.5481
Eye corrosion	-	0.9859
Eye irritation	-	0.9690
Ames mutagenesis	-	0.5200
Human ether-a-go-go inhibition	+	0.6964
micronuclear	+	0.7300
Hepatotoxicity	+	0.5250
Acute Oral Toxicity (c)	III	0.4510
Estrogen receptor binding	+	0.6462
Androgen receptor binding	-	0.6443
Thyroid receptor binding	+	0.6964
Glucocorticoid receptor binding	+	0.5370
Aromatase binding	-	0.4860
PPAR gamma	+	0.5498

+ = positive to specified ADMET classification, - = negative to specified ADMET classification, Acute Oral Toxicity catergory III = median lethal dose of compound ranges between > 500 mg/kg to < 5000 mg/kg.

Table S11. Tabulated toxicity prediction results of compounds **3** obtained from the web-based prediction tool admetSAR.

**ADMET predicted profile classification**	**Prediction**	**Probability**
Human Intestinal Absorption	+	0.9848
Caco-2	-	0.7112
Blood Brain Barrier	+	0.9810
Human oral bioavailability	+	0.6571
Subcellular localization	Plasma membrane	0.4395
OATP2B1 inhibitor	-	1.0000
OATP1B1 inhibitor	+	0.9271
OATP1B3 inhibitor	+	0.9378
MATE1 inhibitor	-	0.8600
OCT2 inhibitor	+	0.5250
BSEP inhibitor	+	0.6706
P-glycoprotein inhibitor	+	0.5812
P-glycoprotein substrate	-	0.5308
CYP3A4 substrate	+	0.6437
CYP2C9 substrate	-	1.0000
CYP2D6 substrate	-	0.8369
CYP3A4 inhibition	+	0.8992
CYP2C9 inhibition	+	0.5640
CYP2C19 inhibition	+	0.8192
CYP2D6 inhibition	-	0.7768
CYP1A2 inhibition	-	0.5542
CYP inhibitory promiscuity	+	0.9563
UGT catalyzed	-	0.0000
Carcinogenicity (binary)	-	0.9429
Carcinogenicity (trinary)	Non-required	0.5249
Eye corrosion	-	0.9838
Eye irritation	-	0.9516
Ames mutagenesis	-	0.5500
Human ether-a-go-go inhibition	+	0.7735
micronuclear	+	0.8100
Hepatotoxicity	+	0.5750
Acute Oral Toxicity (c)	III	0.5169
Estrogen receptor binding	+	0.5955
Androgen receptor binding	-	0.6220
Thyroid receptor binding	+	0.5870
Glucocorticoid receptor binding	-	0.5065
Aromatase binding	-	0.4866
PPAR gamma	+	0.5750

+ = positive to specified ADMET classification, - = negative to specified ADMET classification, Acute Oral Toxicity catergory III = median lethal dose of compound ranges between > 500 mg/kg to < 5000 mg/kg.

Figure S11. Visual representation of the predicted toxicity results of compound **1** obtained from the online toxicity prediction tool STopTox.

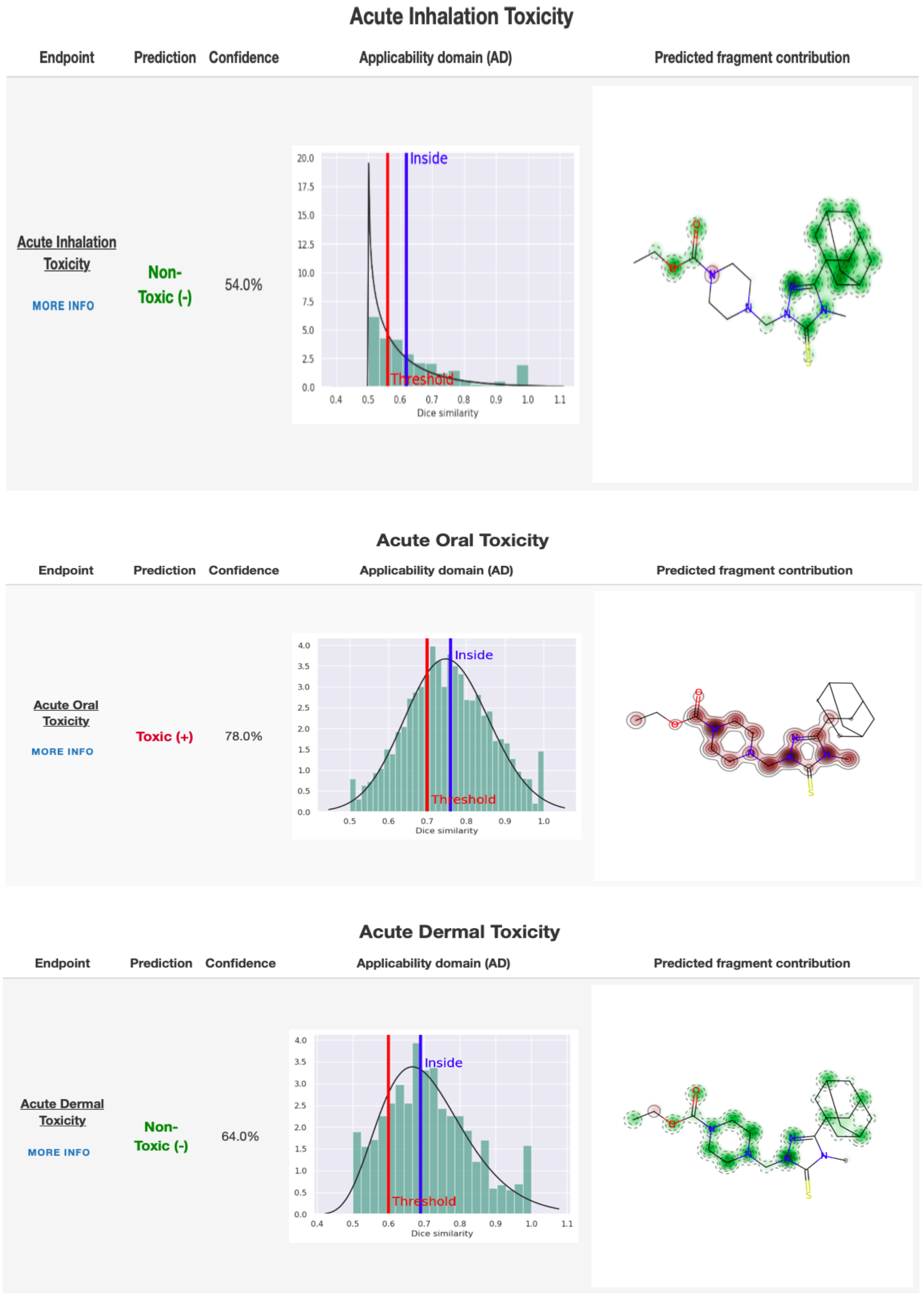

Eye Irritation and Corrosion

Endpoint	Prediction	Confidence	Applicability domain (AD)	Predicted fragment contribution
Eye Irritation and Corrosion<br>MORE INFO	Toxic (+)	71.0%	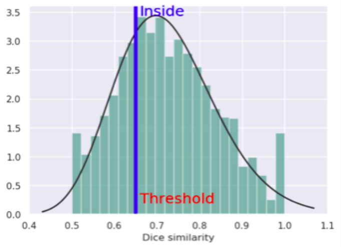	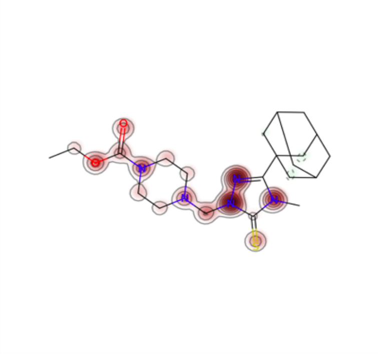

Skin Sensitization

Endpoint	Prediction	Confidence	Applicability domain (AD)	Predicted fragment contribution
Skin Sensitization<br>MORE INFO	Non-Sensitizer (-)	60%	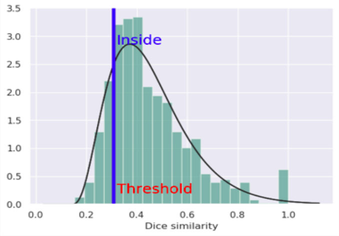	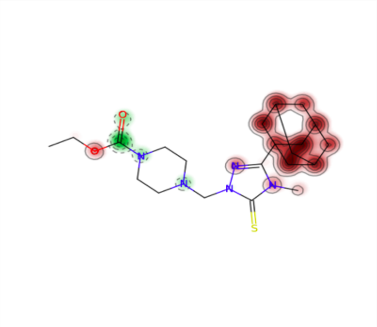

Skin Irritation and Corrosion

Endpoint	Prediction	Confidence	Applicability domain (AD)	Predicted fragment contribution
Skin Irritation and Corrosion<br>MORE INFO	Negative (-)	60%	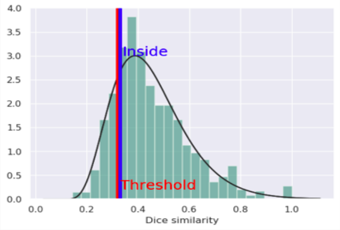	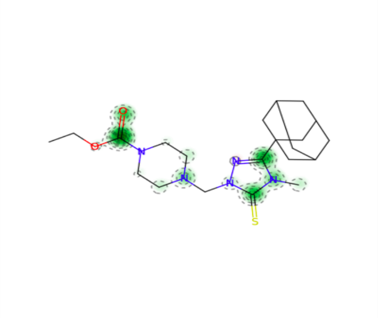

Figure S12. Visual representation of the predicted toxicity results of compound **2** obtained from the online toxicity prediction tool STopTox.

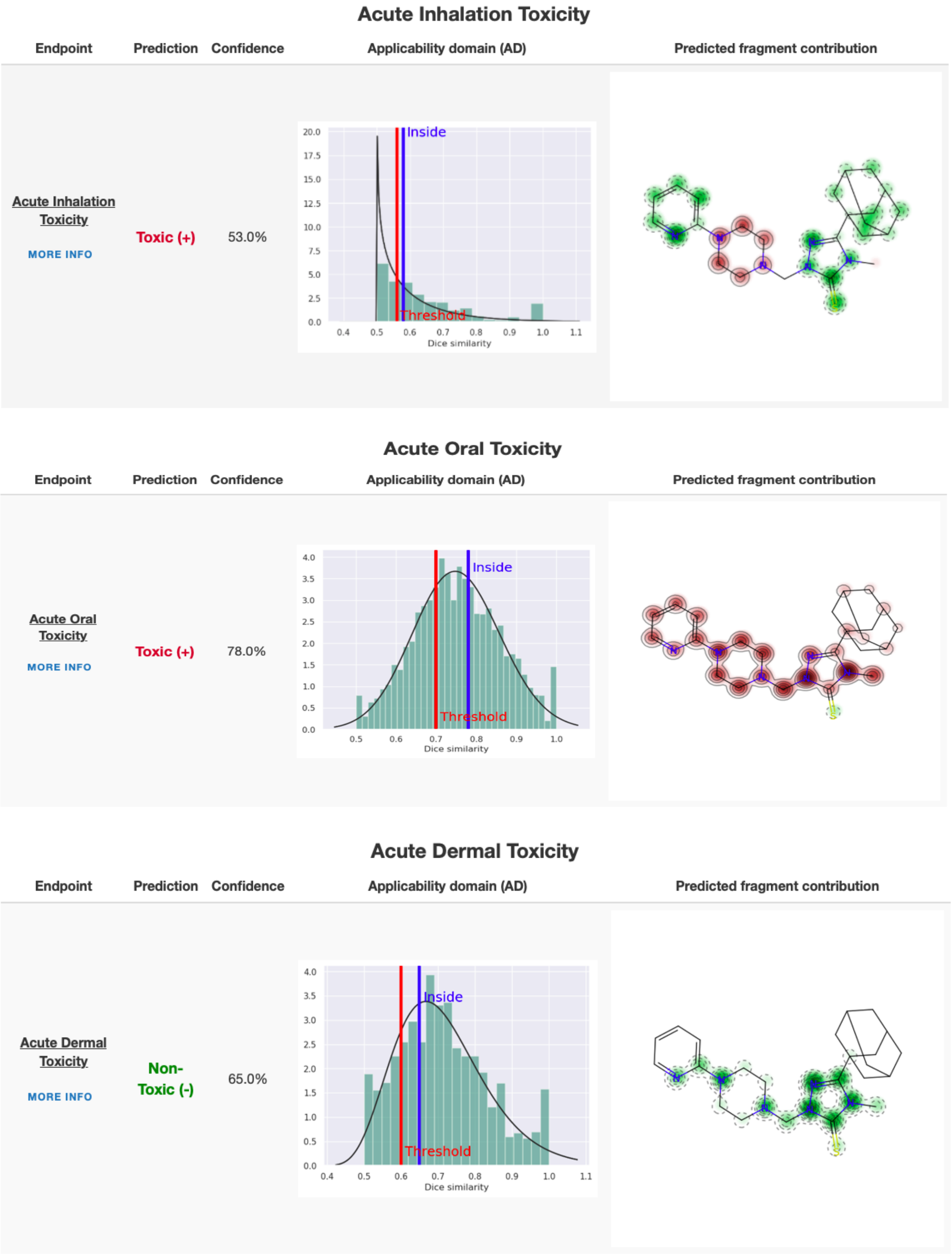

Eye Irritation and Corrosion

Endpoint	Prediction	Confidence	Applicability domain (AD)	Predicted fragment contribution
Eye Irritation and Corrosion<br>MORE INFO	Toxic (+)	54.0%	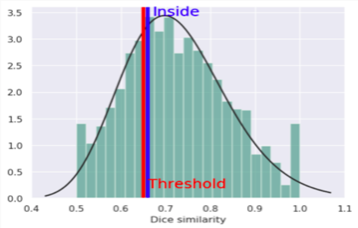	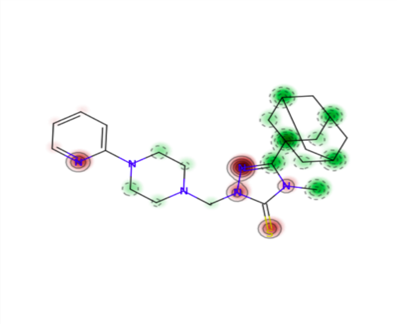

Skin Sensitization

Endpoint	Prediction	Confidence	Applicability domain (AD)	Predicted fragment contribution
Skin Sensitization<br>MORE INFO	Non-Sensitizer (-)	60%	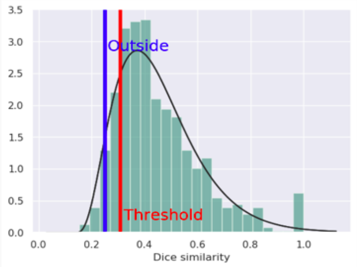	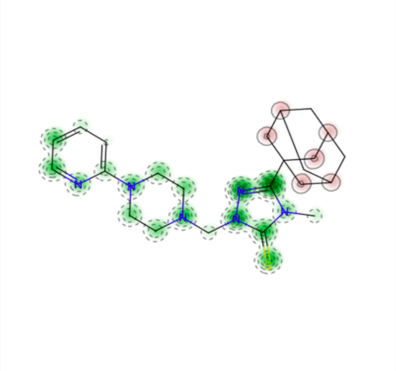

Skin Irritation and Corrosion

Endpoint	Prediction	Confidence	Applicability domain (AD)	Predicted fragment contribution
Skin Irritation and Corrosion<br>MORE INFO	Positive (+)	50%	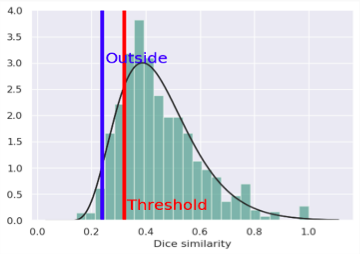	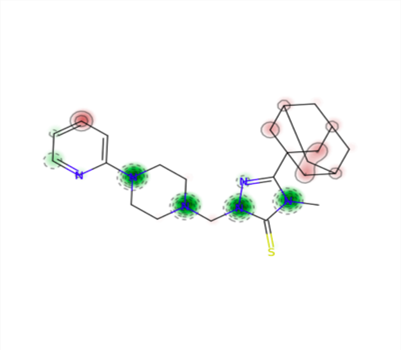

Figure S13. Visual representation of the predicted toxicity results of compound **3** obtained from the online toxicity prediction tool STopTox.

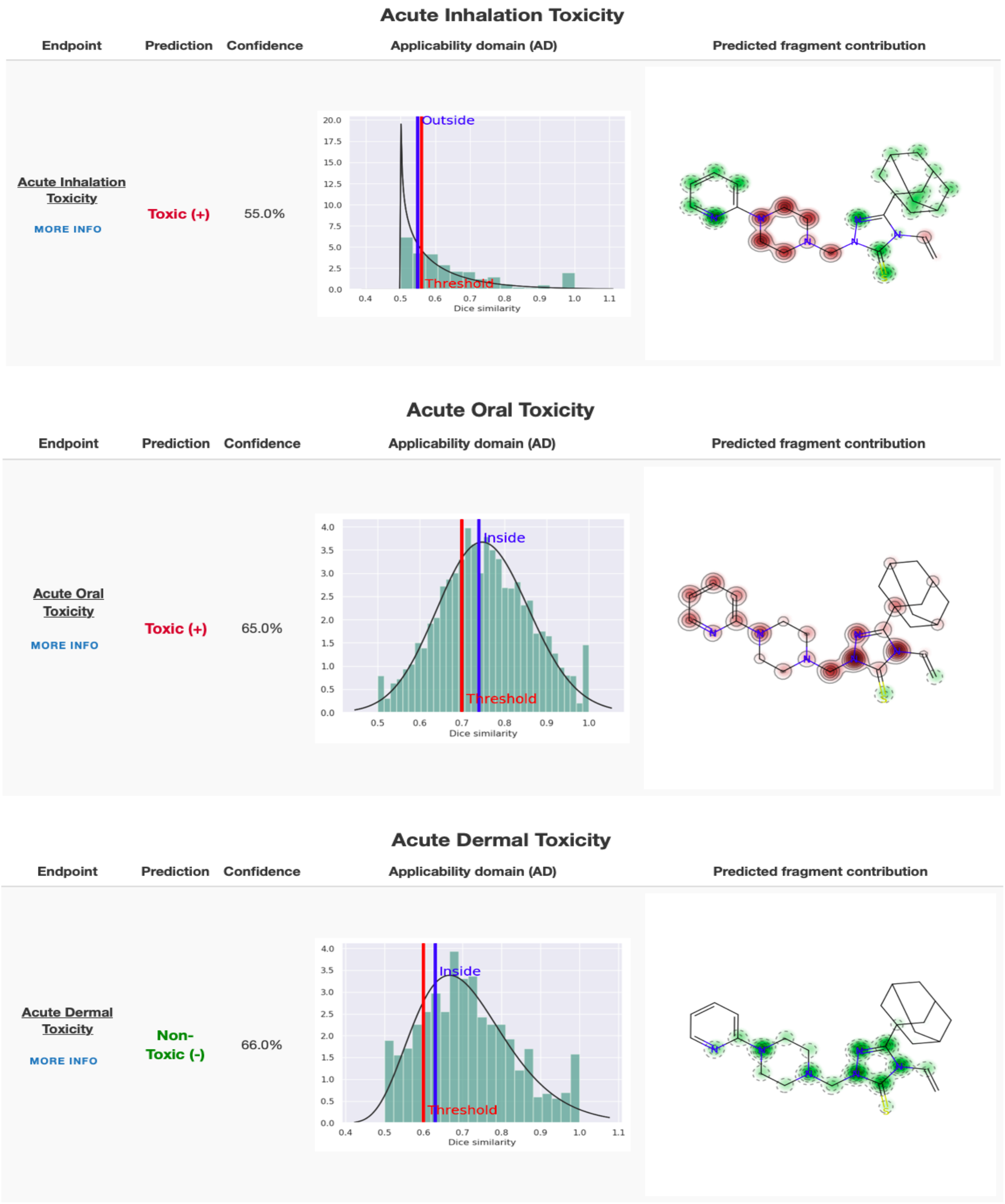

Eye Irritation and Corrosion

Endpoint	Prediction	Confidence	Applicability domain (AD)	Predicted fragment contribution
Eye Irritation and Corrosion<br>MORE INFO	Toxic (+)	62.0%	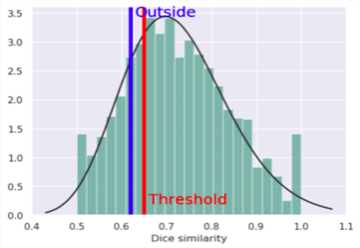	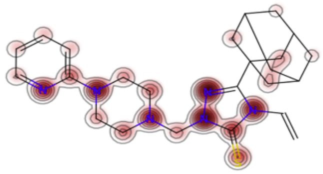

Skin Sensitization

Endpoint	Prediction	Confidence	Applicability domain (AD)	Predicted fragment contribution
Skin Sensitization<br>MORE INFO	Sensitizer (+)	60%	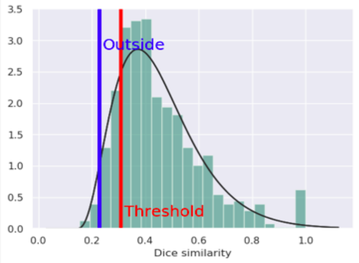	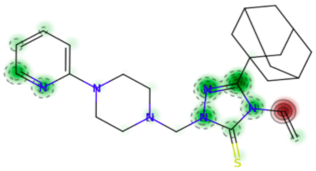

Skin Irritation and Corrosion

Endpoint	Prediction	Confidence	Applicability domain (AD)	Predicted fragment contribution
Skin Irritation and Corrosion<br>MORE INFO	Positive (+)	50%	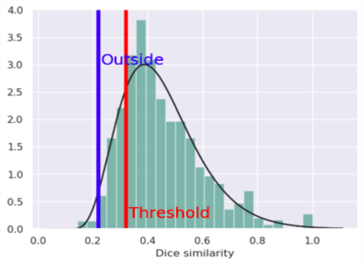	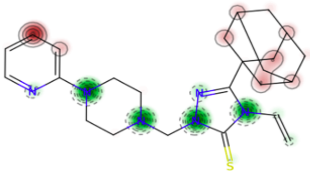
